# Measuring global migration flows using online data

**DOI:** 10.1073/pnas.2409418122

**Published:** 2025-04-29

**Authors:** Guanghua Chi, Guy J. Abel, Drew Johnston, Eugenia Giraudy, Mike Bailey

**Affiliations:** ^a^Meta, Menlo Park, CA 94025; ^b^Department of Sociology, Faculty of Social Sciences, University of Hong Kong, Hong Kong Special Administrative Regions, China; ^c^International Institute for Applied Systems Analysis, Laxenburg 2361, Austria; ^d^Department of Economics, Harvard University, Cambridge, MA 02138

**Keywords:** migration flow, international migration, human migration

## Abstract

Existing estimates of human migration are limited in their scope, reliability, and timeliness, prompting the United Nations and the Global Compact on Migration to call for improved data collection. Using privacy protected records from three billion Facebook users, we estimate country-to-country migration flows at monthly granularity for 181 countries, accounting for selection into Facebook usage. Our estimates closely match high-quality measures of migration where available but can be produced nearly worldwide and with less delay than alternative methods. We estimate that 39.1 million people migrated internationally in 2022 (0.63% of the population of the countries in our sample). Migration flows significantly changed during the COVID-19 pandemic, decreasing by 64% before rebounding in 2022 to a pace 24% above the precrisis rate. We also find that migration from Ukraine increased tenfold in the wake of the Russian invasion. To support research and policy interventions, we release these estimates publicly through the Humanitarian Data Exchange.

## Significance

This paper introduces an approach to measuring international migration, improving on existing efforts in both breadth and resolution. Despite the importance of these figures to researchers, policymakers, and the public, most countries do not report migration flows, and estimates where they exist are often outdated. The International Organization for Migration argues that improved migration data would be highly beneficial, with a value of $35 billion to governments alone. Our estimates improve substantially on existing figures, allowing us to estimate monthly migration flows between 181 countries by drawing on data from more than 3 billion individuals. We are collaborating with NGOs on humanitarian applications of these data and release them publicly to aid researchers and policymakers.

Estimates of migration flows are widely used in evidence-based policymaking, informing efforts to address domestic labor shortages (1), mitigate the negative effects of emigration (2), and increase immigrants’ employment rates (3). Despite the value of these estimates, the International Organization for Migration (IOM) noted in its 2022 World Migration Report that only 45 governments provide data on migration flows, in part because the collection of accurate figures is “extremely difficult” (4, 5). The report also noted that these figures use inconsistent methodologies and definitions of migration and are often out of date: the latest estimates cataloged by the United Nations Population Division date to 2015, while figures for a smaller group of wealthy countries are published only after a three-year delay (6, 7). As a result, the collection of accurate migration statistics was listed as the top objective of the 2018 Global Compact for Migration, with the IOM estimating that better estimates could be worth as much as $35 billion to governments and migrants (8, 9).

To address the limitations of the existing data, researchers have used alternative methods to estimate the rate of migration between countries. One such approach estimates migration flows using time-series data on migrant stocks from surveys or censuses. Though most countries produce data on migrant stocks, such data are usually collected infrequently, making it impossible to calculate monthly migration flows (10–13). Recently, researchers have used new data sources, such as cell phone records and geotagged Tweets, to measure migration in a more timely fashion, but these approaches have been limited in their geographic scope or representativeness (14–20).

In this paper, we estimate migration flows between 181 countries^*^ for each month from January 2019 through December 2022 using privacy protected data from 3 billion active users of Facebook, the world’s largest social network. To do this, we identify changes in the predicted home location of each user over time. We define a migration event as an instance in which an individual who has resided in a country for more than a year moves to another country, which they then reside in for more than one year. This approach matches the United Nations’ recommended definition of migration.^†^ If individuals or families who leave a country and go from place to place in transit (staying less than 12 mo) until they reach the destination, they are not considered migrants to those countries in transit. They are only counted in the migration estimates when they remain in the destination for at least 12 mo. We then aggregate these individual migration events to country-by-country-by-month counts and apply weights to make our estimates representative of the actual migration flows at the population level, accounting for different Facebook usage and levels of economic development along each migration corridor. We validate our estimates against administrative data, which are currently the most reliable sources of information about migration patterns. We find that our figures are strongly correlated with these traditional data sources, where they exist. We release the migration estimates publicly through the Humanitarian Data Exchange (https://data.humdata.org/dataset/international-migration-flows) as a resource for researchers and policymakers. It contains the monthly estimates for each country pair from 2019 to 2022. The list of countries is in *SI Appendix*, Table S9. For each country in the dataset, we release the flow for both directions.

## Global Migration Patterns

**Fig. 1.**
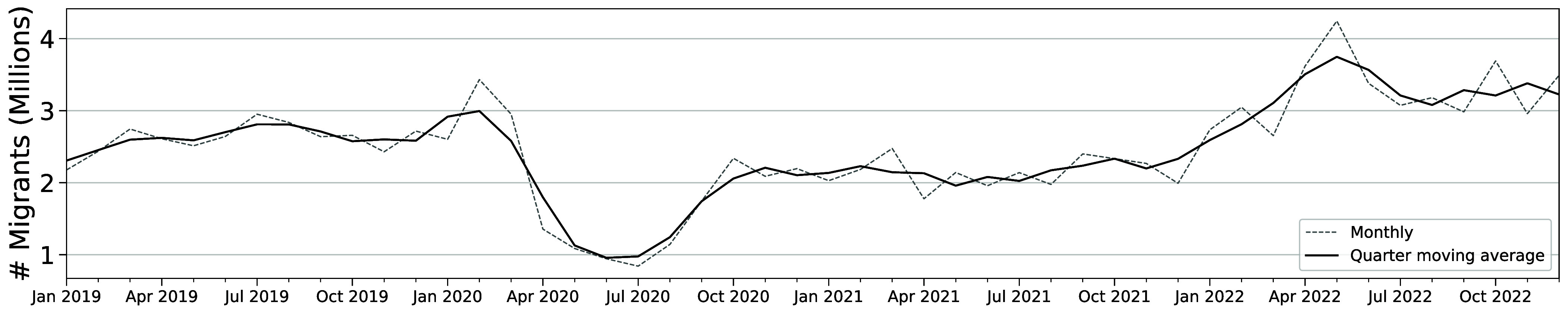
Global international migration over time. The dashed line depicts the estimated level of migration in each month, following the procedure described in *SI Appendix*, *Algorithm* to detect migrants using the selection rate weights described in *SI Appendix*, *Weighting*. The solid line smooths the data over a 3-mo window with 1 mo on each side of the current month.

**Fig. 2.**
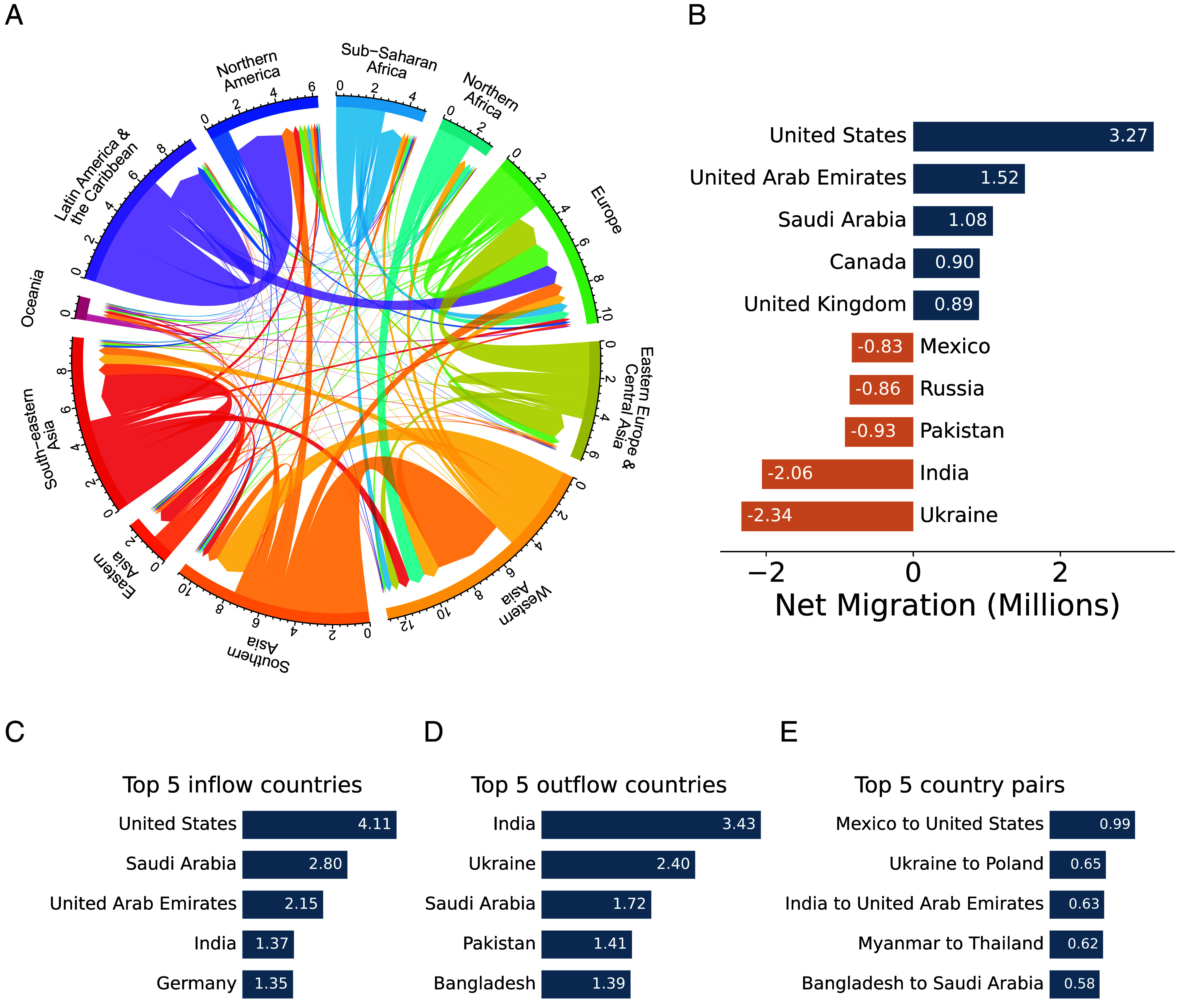
Estimated international migration flows in 2022, in millions of people. (*A*) International migration flows between and within regions in 2022. Lines measure aggregate flows between all country pairs in each region. (*B*) Top and bottom five countries by annual net migration in 2022. Orange bars depict countries with net emigration; blue bars depict those with net immigration. (*C*) Top five countries by annual gross migration inflows in 2022. (*D*) Top five countries by annual gross migration outflows in 2022. (*E*) Top five (directed) country pairs for annual migration in 2022.

We estimate that around 3.3 million people^‡^ migrated each month in 2022 between the 181 countries^§^ in our study (Fig. 1). In total, we estimate that 39.1 million people migrated internationally that year, approximately 0.63% of the population of the countries in our sample.^¶^ The United States had the highest positive net migration in 2022, with over 3.27 million more people migrating to the country than leaving it (Fig. 2*B*). Ukraine saw the largest net outflow, losing over 2.34 million people in 2022 after it was invaded by Russia.^#^ The United States, Saudi Arabia, and the United Arab Emirates are the top three countries in terms of the migration inflows (Fig. 2*C*), while for the migration outflows, the top three countries are India, Ukraine, and Saudi Arabia (Fig. 2*D*).

In Fig. 2*A*, we plot the geographic distribution of these flows at the regional level. This plot displays migration in 2022, though we find broadly similar patterns of migration corridors in the other years we study.^‖^ At the country pair level, Fig. 2*E* shows that close to one million people migrated from Mexico to the United States in 2022. In addition to country pairs with close proximity where migration corridors are prevalent, we also observe the existence of persistent migration between a number of far-away country pairs. To explain this persistence, we next discuss several factors which drive migration along these established corridors.

Distance is a strong predictor of the rate of migration between countries: over 20.9% of migration in 2022 occurred between bordering countries, despite fewer than 1.7% of country pairs sharing a border. Even among countries that do not share a border, we observe substantial clustering of migration at the regional level, with particularly large flows of migrants within Europe, in part due to the lack of internal migration restrictions within the European Union. We also observe notably large flows within Latin America and the Caribbean, driven by migrants from Venezuela to nearby countries.

We also observe several long-distance migration corridors that see persistently high levels of migration. For instance, we observe large flows between West Asia and South Asia, driven largely by labor migration from India, Pakistan, and Bangladesh to Saudi Arabia, Qatar, and the United Arab Emirates. Unusually, this corridor sees largely symmetric migration patterns, since migrants usually return to their origin at the conclusion of their contract (4). Other destinations, particularly Europe and North America, see inflows from a more diffuse set of regions due to the differing set of institutions governing migration to these regions. In *SI Appendix*, *Social Networks and Migration*, we show that social ties between the origin and destination are strongly predictive of these flows, even between geographically distant country pairs.

We also observe that both origin- and destination-country development are strongly predictive of migration rates, with countries classified as high-income by the World Bank attracting 67% of global migrants (22). Additionally, high-income countries send 33% of the global migrants despite comprising just 19% of the world population, aligning with prior research that migrants tend to be wealthier (23). These patterns are visualized in *SI Appendix*, *National Income Levels and Migration Trends*.

Although migration corridors are generally stable, our monthly data allow us to observe how migration patterns shift in response to crises or policy changes. Such shifts, despite their importance in policymaking, have historically been hard to observe due to the limited resolution of existing migration estimates. We observe that crises can lead to dramatic changes in migration; 2.3 million people emigrated from Ukraine following Russia’s invasion of the country in February 2022 and settled elsewhere between February and December 2022 for at least a year, a tenfold increase over the prewar emigration rate. We find that Poland, Germany, the Czech Republic, the United States, and the United Kingdom have received the highest number of migrants, which are closely aligned with estimates from the UNHCR^**^ (24), though the Czech Republic and Estonia have received the largest share relative to their population. We estimate that flows from Hong Kong to the United Kingdom increased more than fifteenfold in the wake of Hong Kong’s passage of a contentious security law in June 2020, while migration from Myanmar to Singapore increased more than fivefold in the wake of a coup in the former country in February 2021. We discuss these patterns in more detail in *SI Appendix*, *Crisis-Induced Migration*.

In recent years, global factors such as the COVID-19 pandemic and its associated migration policy responses have also played an important role in shaping migration. Specifically, after the onset of the COVID-19 pandemic, the global flow of international migrants fell 64%, driven in part by the imposition of border controls by many countries (25, 26).^††^ International migration flows started to rebound after July 2020 as countries began to lift controls (25). Migration rates remained low in 2021, with an average of 2.14 million people migrating each month, 18% below the 2019 average. Migration reached prepandemic levels for the first time in January 2022 and remained high throughout the year, with 3.3 million people migrating on average each month, 24% above the rate in 2019.

**Fig. 3.**
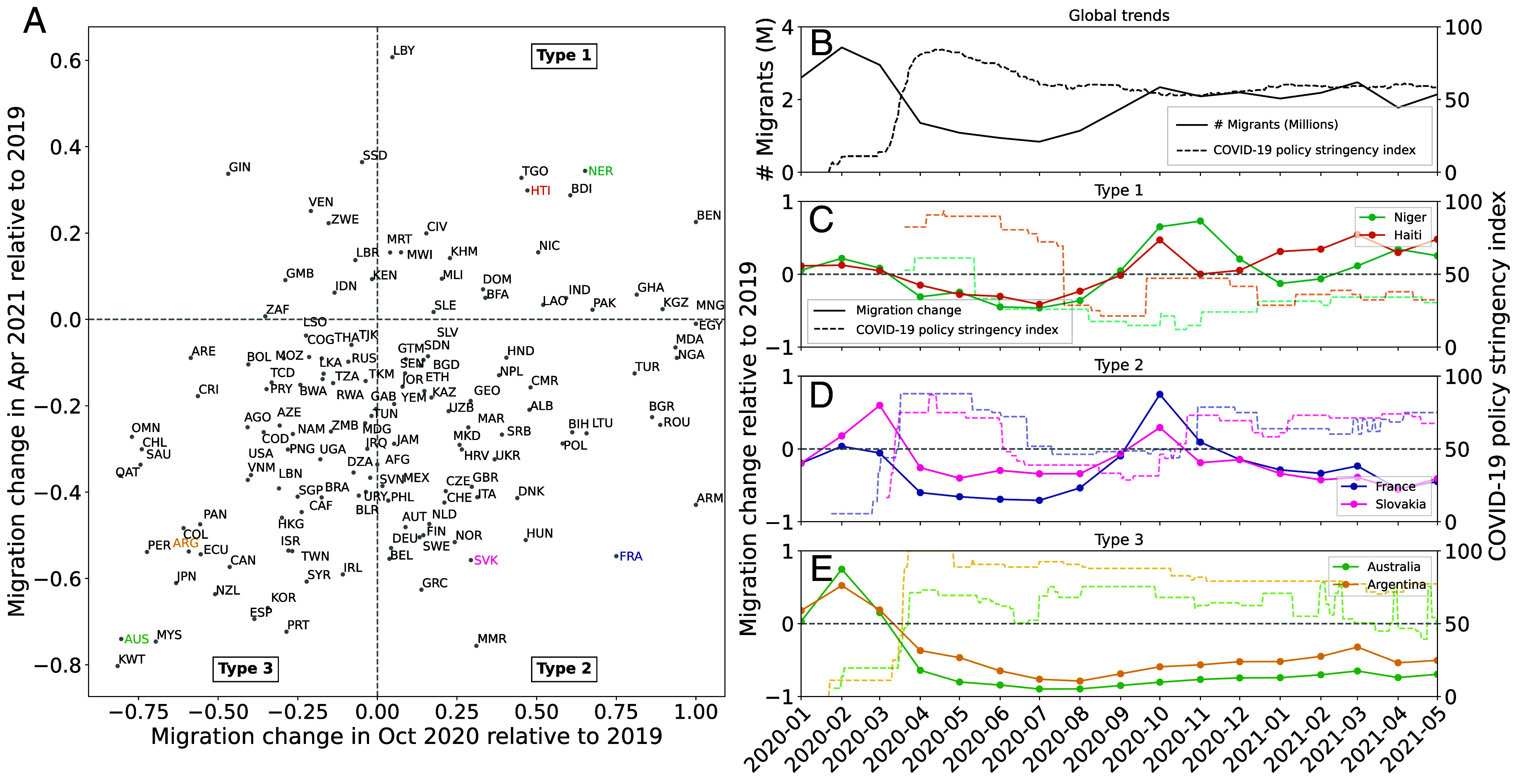
Migration flows and COVID-19 policy stringency. (*A*) Changes in immigration flows during October 2020 and April 2021, relative to each country’s 2019 average. Countries are divided into three types based on their quadrant in this plot. (*B*) Global migration and the mean COVID-19 policy stringency index over time. (*C*) Migration inflows (solid lines) and COVID-19 stringency (dashed line) for Niger and Haiti (two “Type 1” countries where restrictions were relatively low throughout the sample period). (*D*) Migration inflows and COVID-19 stringency for France and Slovakia (two “Type 2” countries where restrictions were relaxed and then tightened). (*E*) Migration inflows and COVID-19 stringency for Australia and Argentina (two “Type 3” countries where restrictions were relatively high throughout the sample period).

Our data also allow us to observe the relationship between COVID-related policies and migration at high frequency. There was a large degree of heterogeneity in national approaches to COVID-19 control; in some countries, the strict border controls enacted in early 2020 remained through 2021, while in other countries controls were never enacted or quickly reversed (Fig. 3). We find that immigration is negatively correlated with the COVID-19 policy stringency index:^‡‡^ a 10-point increase on the 100-point scale is associated with a 5.1% decline in monthly immigrants the following month (27). The COVID-induced drop in migration is more than 50% across all the regions. Migration from South Asia saw the largest decrease in 2020 (dropping 81.5% in 2020 compared to a 2019 baseline), while in North America migration decreased by 55.6% in 2020 relative to 2019.

## Data and Methods

Our first step in estimating migration between countries is detecting a change in a person’s country of residence.^§§^ We determine a predicted country location for each user based on a combination of signals, including the self-reported location on Facebook profiles and the IP addresses used to connect to Facebook. We use a segment-based method to turn these estimates into a time frame of each user’s country location (28). We define migration as living in one country for the majority of a 12-mo period before moving to another country for the majority of the following 12 mo, matching the definition of migration recommended by the United Nations (29).^¶¶^ This algorithm is explained in more detail in *SI Appendix*, Algorithm.

We next aggregate these individual-level migration events to the country-pair level for each month, which provides us with a measure of the number of Facebook users migrating across each corridor. To account for the fact that Facebook usage varies across countries, we weigh our data to make them representative at the population level. After experimenting with alternatives, the weight we apply depends on the country-wide Facebook usage rate and country income level. A single worldwide offset, which is calculated based on the administrative data in New Zealand, is added to control the degree to which the bias of migrants on Facebook varies with development. This method (called selection rate) allows us to account for the fact that in poorer countries, wealthier individuals are more likely to both use Facebook (30) and to migrate. A recent study of selection into emigration found that wealthier individuals were more likely to emigrate in 93 of 99 countries studied (31). We describe the details of this weighting method and compare it with a variety of alternative weighting methodologies in *SI Appendix*, *Weighting*. In *SI Appendix*, Tables S2 and S3, we benchmark our selection model against a naïve approach, in which we weigh migration flows using the inverse the country-wide Facebook usage rate. We find that the selection model reduces the difference between our estimates and those of the Swedish government by about 53.3% relative to the naÃ¯ve approach. We consider a variety of alternative weighting methodologies in *SI Appendix*, *Weighting*. In *SI Appendix*, Fig. S5, we present a country-by-country analysis of the weighted flows and show that the model allows us to account for the fact that selection into both Facebook usage and migration is higher in poor countries. This improves the accuracy of our estimates in the developing world considerably without harming our performance in wealthier countries. We describe our methodology and present further benchmarks in *SI Appendix*, *Weighting*.

After applying the weights, we add noise to our estimates using techniques from the differential privacy literature to preserve the privacy of individual-level data. This generally results in a small amount of noise, with 95% of monthly estimates changing by fewer than seven people. We describe our methodology in more detail in *SI Appendix*, *Differential Privacy*.

## Validation

We next benchmark our estimates against administrative datasets drawn from statistical offices of New Zealand and the European Union. These two datasets are thought to be of unusually high quality, but only capture migration flows to a subset of countries (32, 33). In *SI Appendix*, *Validation*, we benchmark our data against other administrative records, as well as against low-frequency estimates of global migration.

We first benchmark our data against the immigration statistics reported by the government of New Zealand (34). New Zealand has a strong statistical infrastructure and is one of the only countries to release monthly estimates of migration, allowing us to validate both the magnitude and temporal pattern of migration in our data. The New Zealand government uses a nonstandard definition of migration in its statistics, requiring an individual to reside in the country for 12 mo in a 16 mo period to be a migrant, rather than the 6 mo in a 12 mo period used by most nations. In the validation exercises we present in this section, we produced figures using the New Zealand government’s definition of migration, though we use the United Nations definition of migration in our public data release and in all other figures in this paper which include data from New Zealand.

**Fig. 4.**
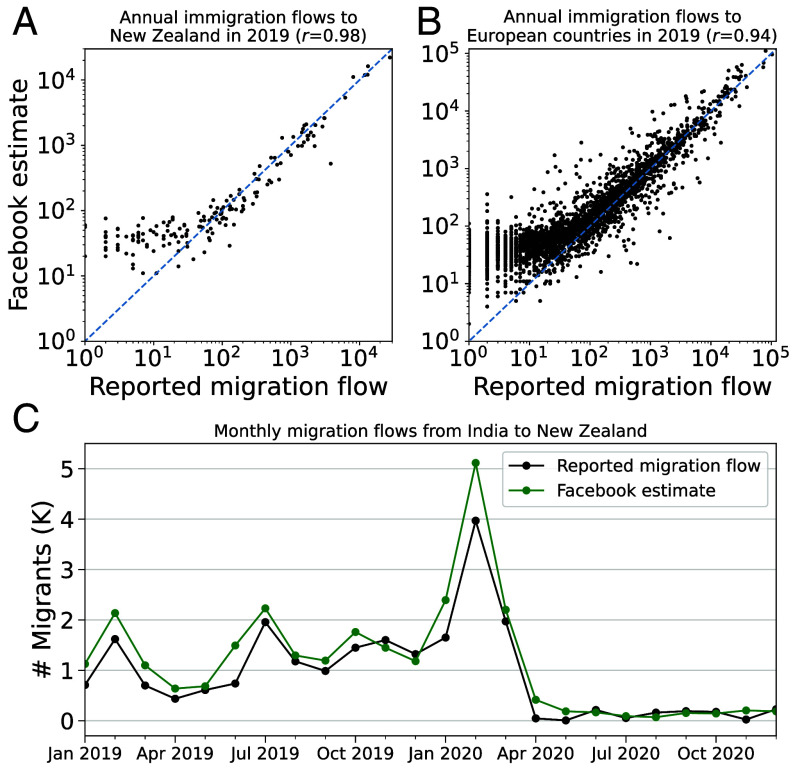
Reported country-to-country migration flows vs. Facebook estimates in thousands. (*A*) Validation of Facebook estimates against 2019 annual data from the New Zealand Statistical Office. Each point stands for the number of immigrants from a country of origin to New Zealand in 2019. (*B*) Validation of Facebook estimates against 2019 annual data from Eurostat, the statistical office of the European Union. Each point stands for the number of immigrants from a country of origin to one European country in 2019. (*C*) Validation of Facebook estimates of monthly migration from India to New Zealand using data from the New Zealand Statistical Office.

In Fig. 4*A*, we compare our 2019 estimates of migration from each country to New Zealand against the government’s official figures. The two series are closely aligned, with similar magnitudes and a correlation of 0.98. Note that our estimates are systematically larger for smaller estimated flows compared to the corresponding reported flows. This is in part because in the differential privacy step, if the number of migrants between a pair of countries becomes negative after adding this noise for each month, we censor the data at 0. After adding those noise annually, our estimates will be consistently greater than our raw estimates. In Fig. 4*C*, we consider the time series dimension of the data, focusing on migration flows to New Zealand from India. Our estimates of monthly migration align closely with the official figures, picking up the spikes in Indian migration in February 2019 and February 2020, at the beginning of the academic year for tertiary education in New Zealand. In 2019, around 13,295 Indian students were pursuing government-funded tertiary education in New Zealand. This was the second-largest group of international students in the country (35). Time series plots of migration from other countries to New Zealand are available in *SI Appendix*, Fig. S8. We also observe that our data capture the dramatic decrease in migration that followed the government’s almost-total closure of the border in March 2020, in response to the onset of COVID-19 (36).

We next benchmark our data against the 2019 migration estimates provided by Eurostat, the statistical office of the European Union. Relative to the data from New Zealand, the Eurostat data are broad in scope, with information on inflows into 22 countries in Europe. The Eurostat data do have several limitations; they are only available at an annual granularity and are collected inconsistently across countries, making use of different methodologies and definitions of migration. We discuss these considerations in more detail in *SI Appendix*, *Validation*. Nevertheless, we show in Fig. 4*B* that our estimates are in general aligned with the figures provided by Eurostat, with a correlation of 0.94 and generally similar levels of migration.

In *SI Appendix*, *Validation*, we explore these benchmarks in greater detail, highlighting several instances in which our estimates diverge from the official statistics. In some cases, these discrepancies are driven by outliers in our model of the relationship between Facebook usage, income, and migration. For instance, we underestimate migration between Samoa and New Zealand, which is driven by a unique policy that allows for an unusually wide swath of Samoans to migrate.^##^ On other migration corridors, we find that our data seem to capture migration excluded from administrative statistics. For instance, the figures reported by Slovakia to Eurostat include only migrants who receive a permanent residency permit, rather than all those who meet the United Nations’ definition of migration. In general, however, we find that our estimates are aligned with administrative figures.

On the whole, these validation exercises highlight the contribution of our estimates relative to existing measures of migration. Our figures generally match existing data where available, but cover a wider fraction of the world and provide finer time granularity at the monthly level. These data provide a strong basis for future research and policymaking related to the causes and consequences of migration.

## Supplementary Material

Appendix 01 (PDF)

## Data Availability

Some study data are available. The individual-level data used to construct these estimates are not publicly available because of restrictions by the data provider. Replication materials, which include the scripts to generate the plots in the paper have been deposited in Harvard Dataverse (https://doi.org/10.7910/DVN/LPA925) (38). Anonymized aggregated data and global migration flow estimates data have been deposited in Humanitarian Data Exchange (https://data.humdata.org/dataset/international-migration-flows) (39).

## Footnotes

^*^A complete list of included countries can be found in *SI Appendix*, Table S9. We exclude very small countries and countries in which Facebook is banned or where other constraints restrict our estimation. We produce experimental estimates for China in *SI Appendix*, *Estimates for China*. These estimates use a separate methodology and are not included in most of the figures in this paper.

^†^See the definition provided by the Statistics Division of the United Nations Department of Economic and Social Affairs (https://www.iom.int/key-migration-terms).

^‡^The United Nations estimates a global population of 281 million migrants in 2020 (21). Note that this figure is referring to the migration stock population, not the migration flow. The migration stock is a count of persons who are currently not in their country of birth, regardless of when they arrived at their destination country, while we count the number of people who moved between countries in a given year.

^§^The 181 countries in our study account for 79.2% of the global population. In *SI Appendix*, *Estimates for China*, we demonstrate how we can adapt our methodology to estimate migration to and from China, the largest country not included in our main results. When we include China in our estimates, the countries in our sample account for 97.4% of the world population. Estimates for China are not included in our public data release.

^¶^In *SI Appendix*, *Estimates for China*, we estimate that in 2022 around 81,000 people migrated to China and 1.1 million migrated away, boosting our global migration figures to 40.2 million and 0.53% of the population of the larger 182-country sample.

^#^Our estimate is lower than the number of Ukrainians who sought Temporary Protected Status after leaving the country. We believe this is due to our more conservative definition of migration; see *SI Appendix*, *Crisis-Induced Migration* for more details.

^‖^A regression of the natural logarithm of the number of migrants in each year on year and country-pair fixed effects explains 88.9% of the variation in the data.

^**^See https://data.unhcr.org/en/situations/ukraine for more details. Note that the website shows the cumulative estimates of Ukrainian refugees since 2022, while our estimates show the number of migrants from Ukraine in 2022. We assume that most of the migrants from Ukraine in 2022 are caused by the Ukraine war as shown in *SI Appendix*, Fig. S20.

^††^In *SI Appendix*, Fig. S18, we present time series estimates for China, and find a largely similar pattern. Migration from China fell from an average of 170,000 per month in 2019 to 93,000 in June 2020. Migration to the country fell by a similar amount, from an average of 21,000 per month to 6,000.

^‡‡^This index by the Oxford Coronavirus Government Response Tracker summarizes a number of COVID-19 policies, including international travel controls.

^§§^This research was conducted at Meta and not reviewed by an Institutional Review Board but has been approved via the privacy review process described at https://about.meta.com/privacy-progress/. The Privacy Review section in the link provided outlines the internal privacy expectations, such as purpose limitation, data limitation, and fairness. All analysis in this study was conducted on deidentified data.

^¶¶^Although we follow this recommended definition in our main analyses, it is easy to adapt our approach to use alternative definitions of migration. For instance, in *SI Appendix*, Fig. S3, we produce estimates using a 6-mo threshold for migration, allowing us to capture more temporary labor migration.

^##^This policy, called the Samoan Quota (https://www.immigration.govt.nz/about-us/media-centre/common-topics/samoan-quota-scheme), allows around 0.5% of the Samoan population (and their dependents) to migrate to New Zealand each year. This allocation is unusually large and conducted by lottery, so migrants admitted under this scheme have a different pattern of selection than those admitted under other provisions of New Zealand’s immigration system, which normally favors relatively educated individuals from sending countries (37). The selection of Samoans migrating to New Zealand also contrasts sharply with the global pattern of migration to rich countries, which favors wealthier migrants and which is used to fit our model of selection.
