# Supplementary material for: Measuring global migration flows using online data
Source: Proc Natl Acad Sci U S A. 2025 Apr 29;122(18):e2409418122. doi: 10.1073/pnas.2409418122 (PMC12067262; doi:10.1073/pnas.2409418122)
Supplement: Supplementary file 1 — Appendix 01 (PDF) [file pnas.2409418122.sapp.pdf]

Supporting Information for

Measuring Global Migration Flows using Online Data

Guanghua Chi, Guy Abel, Drew Johnston, Eugenia Giraudy and Mike Bailey

Guanghua Chi, Guy Abel.
E-mail: gchi@meta.com, guyabel@hku.hk

This PDF file includes:

Supporting text
Figs. S1 to S23
Tables S1 to S9
SI References

Supporting Information Text

Materials and methods

Data. In this paper, we use a variety of data sets to produce our migration estimates for Facebook users, to weight our estimates to capture population-level migration, and to validate our figures against external data. In this section, we describe the data we use in each of these processes.

Location assignment. Our estimates of global migration flows are based on aggregated, weighted observations of changes in individual countries of residence for 3 billion monthly active users on the Facebook platform[*]. We currently use data on migration between the start of 2018 to the end of 2023. Since the United Nations definition of migration requires continued residence in the origin and destination for a year, this allows us to produce migration figures for all months between January 2019 and December 2022. We determine the country of residence for each individual using an algorithm that takes into account signals such as the IP addresses they use, their self-reported home country, and their activity on the platform. The same predictive model for home country location has been used in other studies based on active users of the Facebook platform (1–3). Due to a one-off change in the location prediction algorithm, many users were assigned to a new location in October 2021 and subsequent months. As these changes reflect a shift in methodology, rather than a true change in migration patterns, we impute the number of migrants in October 2021 for each country pair by averaging the level of migration in September 2021 and November 2021. The influence of the home country prediction algorithm change is typically realized within less than a month, which does not have a long-term effect. This means that the change in the algorithm represented a one-time adjustment to certain users' location assignments, rather than an ongoing impact on the process through which moves are imputed. Besides, the location prediction algorithm change resulted in a minimal proportion of users experiencing a change in their predicted home country because the existing algorithm already has a high degree of precision. We drop a small number of other data points, which include 47 country pair-by-day cells, from our dataset when changes in the home prediction algorithm affect individual country pairs before aggregating our data to the country pair-by-month level[†]. We additionally drop a small number of country pair-by-month cells due to data irregularities[‡]. We also omit certain country pairs due to sensitive geographies or countries with disputed borders where we are not sure our estimates would be capturing migrants as opposed to large volumes of other cross border movement.

Population data. As part of the migration estimation process, we use population counts when calculating the weights in each country, as described in SI Weighting section. We use annual population estimates for 2019-2022 from the World Bank[§], which compiles data from the United Nations Population Division, national statistical publications, Eurostat, the United Nations Statistical Division's Population and Vital Statistics Report, and the Secretariat of the Pacific Community. We also use the population data as part of our validation exercises to normalize the immigration counts by the population of the receiving country.

Validation data. We use a variety of alternative migration estimates to validate our data, which are described in more detail in SI Validation. In all the analyses below, we focus on using data on reported immigration, since this is generally thought to be more accurate than data on emigration (4, 5).

In the Validation section and in SI Validation, we make extensive use of migration data from the National Statistical Office (NSO) of New Zealand, which provides monthly migration statistics on its website (6). This data has been widely explored in prior research and is thought to be of comparatively high quality (7). In this context, individuals are counted as migrants if they are "an overseas resident who arrives in New Zealand and cumulatively spends 12 out of the next 16 months in New Zealand." This contrasts with the recommendation of the United Nations, which requires migrants to live in their destination for the majority of 12 consecutive months to be considered residents. To account for this difference, we adjust the parameters in our model (see SI Algorithm) to match those used by the New Zealand government when benchmarking our data against theirs. This modification only applies to the benchmarking step; when measuring migration to New Zealand in the body of the paper, we use the United Nations' definition of migration.

In SI Validation, we also make use of data from Sweden, which publishes migration statistics based on the national population register[¶]. Migrants from abroad who intend to reside in Sweden are required to register in order to obtain a personal identification number that is widely used by the government. During the registration process, the previous country of residence is recorded, alongside other details, which allows for the enumeration of annual immigration flow data by country of origin[‖].

In the Validation section and in SI Validation, we also employ data from Eurostat[**], the statistical office of the European Union, which collates data on immigration statistics from various NSOs. Each member nation has developed different systems for collecting and defining migration. Some member countries, such as Sweden and Denmark, base their figures on population registers, while others, such as Ireland and Portugal, employ estimates based on labor market surveys. Consequently, both

[*] See Meta Reports Third Quarter 2022 Results

[†] This represents around 0.0001% of the total country pair-by-day cells in our sample (47 cells / ((181 * 180) country pairs * 1460 days)).

[‡] We omit, in total, 101 country pair by month cells, which comprises 0.006% of total cells (101 / (48 months * (181 * 180) country pairs)). These cells are: Uganda to Syria between May 2019 and September 2019, Syria to Uganda between May 2020 and February 2021, Myanmar to Cambodia between November 2020 and December 2022, Senegal to Guinea-Bissau between January 2019 and July 2020, Timor-Leste to Indonesia between September 2019 and December 2022 and Afghanistan, Argentina, Chad, Turkey, and Uganda to Sudan between January 2019 and February 2019.

[§] See here for the data source

[¶] See Immigrations and emigrations data from Statistics Sweden

[‖] See this report by M. Forsberg for further information.

[**] This data is available on the Eurostat website as series migr_im5prv.

data quality and the exact definition of migration vary across countries. In recent decades the European Union has introduced legal frameworks to harmonize the definitions used by countries in their required reports to Eurostat, though the definitions and methodologies are not yet perfectly compatible[††]. The data covers reported immigration flows to the 27 current members of the EU, along with Iceland, Norway, North Macedonia, Montenegro, Switzerland, and the United Kingdom. Most countries are present in each year, but data are missing in some countries, such as the United Kingdom, which is only present in 2019. For most countries, the immigration data is broken down by the country of origin (which can be a non-Eurostat nation). Some countries only report pairwise data to Eurostat for a subset of origin countries. When validating the total rate of migration to or from a country, we also include migrants whose exact origin is not specified[‡‡].

We also validate our estimates using data from Germany[§§] in a secondary validation exercise discussed later. The international migration flows to Germany are collected by the registration offices in Germany and are available at a monthly level, which makes them an appealing source of validation for our high-frequency estimates of migration. However, the time frame used by the German government to define migration events is much shorter than that recommended by the United Nations. We discuss these differences in more detail in SI Validation.

Finally, we compare our data against global estimates of migration that are imputed using changes in migrant stocks. This data is only available at a five-year granularity so in our comparisons, we compare our estimated level of migration in 2019 against the annual average in the 2015-2020 period (8).

Algorithm for detecting migration events. To determine if users migrate internationally, we search within each individual sequence of home country locations to determine if their home country changes for at least 12 months. This definition aligns closely with that used by the United Nations Statistics Division, which defines a person's usual residence as "the place at which the person has lived continuously for most of the last 12 months (that is, for at least six months and one day), not including temporary absences for holidays or work assignments, or intends to live for at least six months" (UN Statistics Division, 2008, p102).

We assume that all changes of 12 months or longer in the user's home country correspond to changes in their country of residence (i.e., migration events), rather than extended absences for holidays or work assignments, as it is not possible to determine the purpose of any user's change in their home country from signals on Facebook.

Our algorithm for detecting migrant events for each individual is based on Chi et al. (9). The method first detects each user's segments in time when they live in the same location (allowing for small periods in other places due to travel). The country of each segment is a person's country of residence over that period of time. The method then designates them as a migrant if two adjacent segments are in different countries. Assume we detect two segments for a person: a segment in country A from Apr. 1, 2019 to May 1, 2020 and a segment in country B from May 2, 2020 to Dec. 1, 2024. Then this person is a migrant who migrated in May 2020 from country A to country B. This person is a resident of country A from Apr. 1, 2019 to May 1, 2020, and a resident of country B from May 2, 2020 to Dec. 1, 2024.

To detect segments, we define the maximum gap between consecutive days ϵ. A segment of time in a given country will be considered continuous if there are no periods of more than ϵ days in that period in which the user is seen only outside the country. Detected segments might have different lengths and might have different proportions of days when a person lives in the country of residence. To ensure that the detected segments meet the length of residency and that a person lives there for most of their time, two additional parameters are used by the algorithm for 1) the minimum length of the segment $minDays$ and 2) the proportion of days in each segment $propDays$ (Fig. S1). We set each of these parameters to match the definition of long-term migration recommended by the United Nations. We set the segment length to at least 12 months and the proportion of days in each segment to 50%. As the United Nations does not provide a recommendation of the maximum gap between consecutive days in the destination country (referred to as the radius), we set this parameter to 60 days (see SI Alternative radii for details). In some cases, gaps between the last day of a segment and the start of the subsequent segment are visible. We drop individuals from the estimation if there is a gap of longer than 60 days due to uncertainty about the migrant's residence during this time. See Chi et al. (9) for a full explanation of the segment-based algorithm to detect migration events.

We explore variations in the parameter values for the algorithm elsewhere in the paper. For instance, when validating our data against the migration data provided by the New Zealand NSO, we set $minDays$ to be 16 months (487 days), and $propDays$ to be 75% (12 months), to match the definition used in their administrative data. We also present results after setting $minDays$ to be 6 months (182 days), and $propDays$ to be 50% (3 months), which allows us to measure more recent migration trends (see SI Alternative minimum segment lengths).

Alternative radii. We considered several factors to inform our decision to set the radius ϵ to be 60 days in our segment-based algorithm. As the value of ϵ increases, it allows users to spend more consecutive days outside of their country of residence during a segment. When ϵ is large, we can better account for users who temporarily travel outside their country of residence, but segments are then more likely to overlap, if, for instance, a user alternates stints in two countries. A smaller radius lessens the number of overlapping segments but imposes stricter requirements on the length of trips that users can take abroad during their residence in a given area.

[††] A full list of methodologies can be found in Section 18 of the description of Eurostat series "migr_immi", at this link.

[‡‡] For more information, see here. Article 3(1)(A)(iii) describes the mandate to collect this data, and Article 9(1) discusses allowable sources and methodologies. Table 18 of this link describes the processes used by each country to produce their immigration estimates.

[§§] See here for data source and here for data details.

To justify our choice of ϵ, we consider three metrics. Our first metric is based on analysing the dominance of the modal country within each segment. Concretely, we measure the percentage of segments in which the modal country within the segment accounts for more than 90% of days in the segment. We also consider an alternative metric which measures the number of segments in which the most common country and second-most common country are almost as frequent as one another, differing by fewer than 20% of the total number of days in the segment.

Our third metric combines the number of transitions and the longitudinal entropy, following the procedure outlined in Gabadinho, Ritschard, Studer, and Müller (10). Specifically, we calculate for any sequence of s:

$$C(s) = \sqrt{\frac{\ell_d(s) - 1}{\ell_s - 1} \frac{h(s)}{h_{max}}}$$

where h_{max} is the theoretical maximum entropy given the cardinality of our set of countries. h_s is the entropy of a sequence s. $\ell_d(s) - 1$ is the number of transitions in a sequence s. $\ell(s) - 1$ is the maximum number of transitions in a sequence s. The complexity index attains its minimum value of 0 when there are no transitions in a sequence and its maximum value when each state in a sequence is different. In other words, if a sequence consists of a single state that is repeated throughout, the complexity index will be 0, whereas if each state in the sequence is unique and different from all the others, the complexity index will be at its maximum.

As shown in Fig. S2, we find that, when we set a lower value of epsilon, migrants' location histories exhibit lower complexity, and there are fewer cases where users split their time nearly equally between two countries during a segment. At the same time, we find that the overall number of detected migrants falls substantially when we set ϵ to lower values, particularly when we set it equal to 30 days. In this case, we drop over 6% of our total number of migrants, as we ignore segments that might include short visits to family, tourist excursions, or business trips. When ϵ is small, the detected segments need to have a very continuous record in a country because the gap between two days when a person lives in the country must be smaller or equal to ϵ. In the extreme, when $\epsilon = 1$, a person must live in the same country every day to form a valid location segment. Consequently, using a smaller ϵ in the algorithm will lead to fewer migration events detected. When ϵ is large two potential issues might arise: (1) the proportion of days ($propDays$) a user is identified in the segment can become smaller as the larger ϵ allows for more gaps between the days a person is located in their country of residence and (2) neighboring segments are more likely to overlap, which will be dropped in our algorithm. The remaining segments after dropping the overlaps might be shorter than 12 months, and hence will no longer be valid for detecting migration events based on the UN definition. These two issues result in fewer migrant events detected when using larger ϵ, and hence we choose to set ϵ to equal 60 days to balance these considerations.

Alternative minimum segment lengths. Estimates of international migration are typically available only with a considerable time lag for publication. Our estimates rely on digital trace data that is gathered in near real-time. As we adopt the United Nations' recommended definition of migration, which requires migrants to reside in their destination for the majority of a year, we need to wait until the completion of 12 months to detect migration events for the past year. In this section, we explore an alternative definition of migration which could allow us to measure global migration with a shorter time lag, potentially opening up new opportunities for research and policy.

To create timelier estimates, we consider a different definition for residence, in which a migrant still needs to be a resident of their origin region for more than six months in a 12-month period, but the required duration in their destination is shorter–only 3 months in a 6-month span. This change allows us to capture migration with only a 6-month lag, at the cost of capturing some migration that does not match the United Nations' definition. As shown in Fig. S3, using this shorter definition, we detect slightly more migrants than we do when applying the United Nations' definition due to our inclusion of shorter-term migrants, which is consistent with the findings of Nowok and Willekens (11).

In Fig. S4, we correlate our estimated migration levels in 2019 using this method against data from Sweden and Eurostat. We can see that, although we tend to slightly overestimate migration with this new definition, we still see a strong relationship between the reported migration statistics, with a Pearson correlation of 0.90 with the Swedish data and 0.86 with the Eurostat data. We do not benchmark the short-term results against New Zealand's data because its definition of migration implies even longer minimum stays than the United Nations definition.

Comparison with frequency-based method. The frequency-based approach, in which one's residence is considered to be the location where a user spends the majority of days in a given calendar year, is a common method to detect migration (12–14). In this methodology, an individual is considered a migrant if their modal location changes between calendar years. Prior work has found that the segment-based method has a better performance than the frequency-based method based on human-labelled data (9), but these two methods have not been previously compared in the context of the digital trace data that we use in this paper. In this section, we benchmark our preferred segment-based method against a frequency-based method and highlight how the two methods map onto the United Nations' preferred definition of migration.

Although the modal country is measured annually with the frequency-based methodology, this approach does not ensure that a migrant lives primarily in a country for an entire year. Correspondingly, the frequency-based definition classifies a number of short-term moves as migration events. In Table S1, we show that 18.7% of moves under the frequency-based method have a duration of fewer than 300 days, which are not classified as migration events in the segment-based definition of migration or the UN definition. The frequency-based approach also diverges from the United Nations' definition of migration and from the segment-based approach as it does not impose any constraints on the minimum number of days that an individual must be

present in their destination during the residence period. As a result, we find that the frequency-based definition detects many migration events for individuals who frequently move between locations, even though these short-term moves are not captured by the segment-based approach or by the United Nations' definition of migration.

In Table S1 we demonstrate that using the segment-based definition, 97.8% of the individuals spend 90% or more of their residence period in the country assigned to them. In the frequency-based approach, individuals only spend 70.5% of their time in their assigned country of residence. We also find that the frequency-based method leads to a far higher number of cases in which individuals split their time evenly between two countries, but are still recorded as migrants. As a result of these factors, we find that the frequency-based method assigns users more complex and less continuous migration histories.

The frequency-based approach also has another additional drawback relative to the segment-based approach, namely, it does not neatly capture the date on which a migrant moved. For example, for a person whose modal home country changed from one country in 2019 to another in 2020, it is unclear if the person migrated in 2019 or 2020. This issue makes it harder to directly compare the migration estimates from the frequency-based technique with those provided by administrative sources. Nevertheless, we can still get a sense of how our migration estimates using this method compare to administrative data by comparing them to both of the neighboring years. For instance, we can compare our frequency-based migration estimates for 2018-2019 and 2019-2020 against the Eurostat migration estimates from 2019 at the country-pair level, finding Pearson correlations of 0.79 and 0.86, respectively. These results compare unfavourably with those from our segment-based method in 2019, which had a correlation of 0.93 with the Eurostat estimates, see SI Validation for full details on our validation exercises.

In summary, we find the segment-based approach performs better than traditional frequency-based methods for detecting international migration events in four aspects: (1) individuals spend a higher proportion of their time living in their detected county of residence, (2) individuals have fewer transitions across countries, (3) the precise timing of migration events are known, and (4) comparisons with validation data are facilitated by the use of more-similar definitions of migration.

Weighting. We observe migration decisions only for individuals who actively use Facebook. In order to provide estimates for the total population level of migration, including migration among non-users, we adjust our initial estimates using a variety of weighting mechanisms. An ideal weighting mechanism would allow us to account for a variety of (potentially unobserved) factors that affect both Facebook usage rates and migration propensity. In this section, we discuss a range of weighting methods used in migration estimation. To benchmark these methods, we use each one to weight the level of migration we observe among Facebook users, and benchmark these series against administrative records documenting the number of individuals arriving in Sweden during each year between 2019 and 2022. Sweden's migration data is derived from the country's mandatory population register and is considered to be of high quality, as it is available at the country-pair level and captures essentially all long-term stays in the country, regardless of visa type (15).

Raw estimates. We first benchmark our initial estimates against the Swedish administrative data, calculating both the Pearson correlation between the two series and the sum of the absolute errors. As shown in the third column of Table S2, the raw estimates are highly correlated with the Swedish data ($r = 0.84$, 0.93, 0.95, 0.96 from 2019 to 2022)[¶¶]. Though the two series are highly correlated, we can also see that the sum of the distance between the initial estimates and reported migration statistics is relatively high. In Table S3, we show that this is largely because we underestimate the level of immigration from almost all countries since we do not account for the migrants to Sweden that do not use Facebook.

Selection rate. In the main results discussed in the paper, we use a hybrid approach that harnesses several features of the joint relationship between socioeconomic status, migration, and Facebook usage. In Fig. S5, we show that when we weight our raw data using the inverse penetration rates, we obtain relatively accurate estimates for high-income countries, but we tend to dramatically overestimate the rate of migration from lower-income countries. These results indicated that there is variation in the degree of selection into migration and Facebook usage across countries, with less-developed countries tending to have a less representative sample on the platform. This finding aligns with a large literature in economics highlighting the barriers that poor individuals face both in migrating and in accessing communications technology (16, 17). In developed countries, these constraints bind fewer people, and the degree of selection into migration and Facebook usage is likely to be less severe.

With this intuition in mind, we can describe the construction of the weights used in our main specification. In high-income origin countries, we would like our weights to resemble the inverse of the penetration rate in origin country o ($\frac{FBUsers_{o,t}}{Population_{o,t}}$), given that Facebook users in such countries are broadly representative of the broader population in their migration propensity (following the analysis in the first panel of Fig. S5). In lower income origin countries, we have observed that Facebook users tend to be more likely to migrate than the population at large, so we would like our weights to assume that the degree of non-random selection into our sample increases as we consider origin countries with lower incomes. Correspondingly, we would like to place relatively less importance on the penetration rate when weighting the data for these countries. To parameterize this relationship, we define the denominator of our weights as a linear combination of the penetration rate in origin country o and a fitted constant r_t (whose construction we will describe shortly), which captures the degree to which selection into our sample is more severe in low-income countries. The contribution of each term to the overall weights is determined by the per-capita income of each country, scaled such that the highest-income country has a value of 1. Concretely, we can define the weight placed on each migrant from origin o in year t as:

[¶¶] We do not include the correlation plot here because it could be used to infer the penetration rate of Facebook users in each country, which is not public information.

$$W_{o,t} = \frac{1}{\text{Income}_o \times \frac{FBUsers_{o,t}}{Population_{o,t}} + (1 - \text{Income}_o) \times r_t} \quad [1]$$

Here, Income_o is calculated using the 2019 Gross National Income per capita (GNIpc)[***] of country o, using the following formula:

$$\text{Income}_o = \frac{GNIpc_o}{\max(GNIpc_o)} \quad [2]$$

To apply these weights in practice, we need to tune a single parameter for each year, r_t, which controls the degree to which selection into the sample varies with development in year t. To choose the parameter used in the paper, we test a variety of possible values for r_t and benchmark the resulting weighted migration figures against figures from New Zealand's NSO at the country pair level. For each year, we test candidate values of r_t between 0 and 3 using the New Zealand data in steps of 0.01 and select the parameter that minimizes the sum of the absolute errors across all origin countries. We find that the optimal value of r_t is 0.46 in 2019, 0.41 in 2020, 0.46 in 2021, and 0.35 in 2022. We then use these values of r_t to weight our data for all other country pairs in that year. Moreover, while the migration patterns of New Zealand may not be representative for most countries, we observe that a small variation in the r measure results in a minor change in the absolute errors, which further strengthens the validity for calibrating r using New Zealand data. In Fig. S6, we illustrate how the sum of the absolute errors in the Swedish data varies with r_t in each year. The optima we find here are quite similar to the optima estimated with the New Zealand data, hinting at the generalizability of the parameters we estimate. We show in columns 11 and 12 of Table S2 that this weighting method results in the lowest total error rate on the Swedish data for 2019, 2021 and 2022, and on the Eurostat data for all the years in our sample.

In principle, this method could be expanded to allow for r_t to differ according to the level of development of the destination country. This could capture the fact that the degree of selection on socioeconomic characteristics into migration could vary according to the wealth of the destination, even holding fixed one's origin country. Our ability to investigate the sensitivity of r_t to these destination characteristics is limited by the scarcity of high-quality immigration data for less-developed countries. With that said, in Table S4, we highlight the strong performance of this methodology using a second validation set from Eurostat. Though this is not a globally representative dataset, the figures in Table S4 show that the selection rate methodology outlined here performs well across a variety of destination countries whose GDP per capita varies by more than a factor of 10 (from a high of \$113,196 in Luxembourg to \$9,518 in Bulgaria)[†††]. We present more analyses in SI Validation below.

Inverse penetration rate weights. The simplest weighting approach is to weight our initial migration estimate from origin country o to destination d in year t by the inverse of the Facebook penetration rate in the origin country in that year: $W_{o,t} = \frac{Population_{o,t}}{FBUsers_{o,t}}$. Under this approach, we assume that no factors affect both an individual's migration decision and their rate of Facebook usage. In columns five and six of Table S2, we demonstrate the implausibility of this assumption, showing that applying the inverse penetration rate weights increases the absolute level of error in our estimates and decreases the correlation of our figures with the Swedish data.

In Column 5 of Table S3, we present the estimates of the total level of migration to Sweden after applying the inverse penetration rate weights. We see that we now overstate the aggregate level of migration to Sweden, which suggests that individuals we observe on Facebook are more likely than an average person in their country to migrate to Sweden. We show in Fig. S5 that the degree of selection is non-uniform with respect to the income of one's origin country. Among wealthy sending countries, it seems that there is little selection into the sample on the basis of migration likelihood, with the inverse penetration weights causing our estimated flows to closely match the administrative data. However, in less developed countries, we see that Facebook usage appears to be non-random, with the inverse propensity weights causing us to overstate migration several-fold. For this reason, we will now turn our attention to weighting methods that can account for these differential rates of selection.

Raking ratio estimation. In addition to a user's location, we are also able to identify users' age, sex and region within their country. We can use these demographic characteristics to further adjust for the non-random nature of Facebook usage and migration. In particular, we can use these additional details to calculate user weights that account for uneven Facebook usage within a country. We use an iterative proportional fitting method (also known as raking) to create weights for each age by sex by region group in our sample, such that the (weighted) prevalence of each group in our data matches the true distribution. To do this, we use WorldPop estimates of the population of each demographic group in each GADM-1 region[‡‡‡]. The raking process was carried out using the survey package in R (18).

We are only able to calculate weights for subgroups that are observed in both Facebook users and possible global data sets on factors that might impact the variation in Facebook usage patterns. Consequently, we are restricted to considering a relatively narrow set of characteristics in our weighting model, where we are unable to directly target other potentially relevant socioeconomic characteristics. We see in columns 7 and 8 of Table S2 that the raking methodology we employ does not improve the correlation of our data with the Swedish administrative figures. Instead, our correlation decreases and the sum of the absolute errors increases, particularly in low-income countries.

[***] See here for data source

[†††] The Eurostat figures are reported individually by each country and vary somewhat in their collection methods and definitions, so some degree of caution is needed in interpreting these results.

[‡‡‡] GADM-1 regions are a global set of top-level administrative divisions. In the United States, these correspond to states. See here for the data source.

Coefficient. The methods discussed above are appealing because the weights used can be calculated directly from country-level demographic data, which is available globally. However, as we have seen, these methods tend to perform poorly because there is likely to be selection into our sample based on characteristics that are not observed across all countries in both Facebook and administrative datasets. In this section, we demonstrate how we can use additional migration data to help us estimate the parameters of a simple model of selection into our sample.

We use a second set of administrative data from the NSO of New Zealand to estimate this ratio, regressing the government's estimates of the number of arrivals from each country on our raw estimates. We use data from New Zealand since its migration figures are also thought to be of exceptionally high quality (7). We then use the estimated coefficient we find as our weighting factor, which is common across all country pairs. In this model, we assume that we observe a fraction of the true number of migrants between each pair of countries, where this fraction is constant across country pairs and over time.

We present the results on the Swedish arrival data in columns 9 and 10 of Table S2. Since we are solely multiplying all of our estimates by a common factor, the correlation is unchanged, although this methodology slightly improves the overall error rate. This implies that the overall rate of selection into migration is relatively similar across the two destinations when we consider all origin countries. That said, the rate of selection into migration seems to vary according to the characteristics of the sending county (see Fig. S5), which we cannot capture using this single-parameter approach (16).

Differential privacy. We use techniques from the literature on differential privacy to protect information about individuals in the aggregated data we make public. Dwork and Roth (2014) (19) proposed the definition of differential privacy and provided typical mechanisms to achieve differential privacy. A randomized algorithm M is (ϵ, δ)-DP if for any pair of data points, X and Y differing at most one row (e.g., an individual in a dataset), and any event E (i.e. all potential output of M),

$$\Pr[M(X) \in E] \leq e^{\epsilon} \Pr[M(Y) \in E] + \delta$$

Balle and Wang (2018) (20), and Dong, Roth, and Su (2020) (21) recently proposed the approach to inject random noise from the Gaussian distribution to the output, to achieve (ϵ, δ)-DP. A randomized algorithm $M(x) = f(x) + Z$ with $Z \sim \mathcal{N}(0, \sigma^2)$ is (ϵ, δ)-DP if and only if

$$\Phi(\frac{\Delta}{2\sigma} - \frac{\epsilon\sigma}{\Delta}) - e^{\epsilon}\Phi(-\frac{\Delta}{2\sigma} - \frac{\epsilon\sigma}{\Delta}) \leq \delta$$

where Δ is L2 sensitivity, which is defined as the L2 norm $(\sqrt{\sum_{i=1}^{k} v_i^2})$ of the maximum possible difference in all the output aggregates caused by adding or removing one individual. We adopt such an approach as it is easy to implement and the noise magnitude is relatively small. Since an individual can only migrate once per year under the definition we use in this paper, the impact of adding or deleting an individual from our data can only affect the value in any specific cell by at most +1 or -1. Under the assumption that we will collect and release yearly data for a total of 10 years, and we will share the data of the total number of migrants, the number of migrants by age, and the number of migrants by sex, the L2 sensitivity will be $\sqrt{30}$.

We use $\epsilon = 10$, $\delta = 10^{-9}$, $\Delta = \sqrt{30}$, which implies that we should inject random noise drawn from a Gaussian distribution with parameters $(0, \sigma = 3.56)$. Accordingly, in 95% of the cases, we will add or subtract fewer than 7 people to each country-by-country-by-month cell. If the number of migrants between a pair of countries becomes negative after adding this noise, we censor the data at 0.

Validation

New Zealand. In Fig. S7, we compare our estimated rates of migration into New Zealand with the figures reported by the country's NSO. On the X axis, we present the official estimate of the migration rate into the country from each origin. We benchmark this against our estimated level of migration, which is weighted using the selection weighting approach described in SI Weighting[§§§]. We find that our data matches the New Zealand government figures extremely well, with a Pearson correlation above 0.98 each year. These figures remain robust in 2020, 2021 and 2022, despite the onset of the COVID-19 pandemic, which dramatically changed the pattern of migration into New Zealand.

Nevertheless, we do highlight in Fig. S7 several sending countries for which our estimates of migration to New Zealand are less aligned with the official figures, namely Tonga (TO), Samoa (WS), and Vanuatu (VU). These three countries are all categorized by three factors: strong cultural and regional ties to New Zealand, relatively low levels of Facebook usage, and preferential visa programs that make migration to New Zealand accessible to a wider swath of society than it would be without government intervention. For instance, New Zealand administered Samoa before the country's independence and signed a treaty in 1962 allowing for preferential immigration of Samoans under the Samoan Quota (SQ), which led to widespread emigration from the country, which now has 50% of its population living abroad. Similar programs exist in New Zealand for Tongans (under the Pacific Access Category) and Vanuatuans (under a variety of temporary worker programs) (22). These programs highlight a limitation of our selection weight approach—though we are generally able to approximate the rate at which economic selection into Facebook usage and migration coincides, we perform less well for country pairs where policies facilitate or hinder migration for certain groups. Since we fit only a single parameter in the selection weight model to explain migration under facilitated conditions and more traditional conditions (and there are many more cases of the latter), our model performs less well for these unusual cases.

[§§§] The data from New Zealand is based on a slightly different definition of migration than is used elsewhere in the paper. In general, we follow the United Nations definition, in which migrants are defined to be individuals who move to a new region for 12 months, having resided elsewhere for 12 months. In the case of New Zealand, migrants are defined as those who arrive in the country and live there for 12 of the next 16 months (perhaps discontinuously) and who lived in a different country for 12 of the previous 16 months. In all of the analyses presented in this section in which we compare our estimates to the figures from the New Zealand government, we use a set of Facebook estimates that are produced using parameters that match the New Zealand government's definition of migration. We use our baseline definition for migration when comparing our data against the figures produced by other NSOs.

In Fig. S8, we further analyze trends in monthly migration to New Zealand from the top five sending countries. We demonstrate that our data capture the same monthly migration patterns as the administrative data, including seasonal variations and the impact of COVID-19 on inflows.

Sweden. In Fig. S9, we present analogous validation exercises, comparing our migration estimates to the administrative figures from Sweden. We find high Pearson correlations between the two sources of data, ranging from 0.87 in 2019 to 0.97 in 2022. Our estimates also closely align with the Swedish government figures in their magnitudes, indicating we are able to match the absolute as well as relative scale of the migration flow for most sending countries.

As in the case of the New Zealand validation exercises, several outliers stand out in this exercise. In 2019, for instance, our estimates are substantially lower than the Swedish government's figures for flows from Afghanistan and Syria, two countries which saw large outflows of asylum seekers during this period. Here, as in New Zealand, policy decisions made by the receiving government, paired with relatively low rates of Facebook usage in the sending countries (especially in Afghanistan and Eritrea) contributed to an unusual pattern of selection in our sample, one in which migration and Facebook usage are less tightly linked than for most migration corridors. We can see further evidence for this interpretation when we compare the 2019 and 2020 data. In 2020, the Swedish government changed its policy on residence permits, eliminating the presumption of eligibility that most Syrians were able to take advantage of in prior years[¶¶¶]. The following year, we no longer underestimate Syrian migration so substantially, perhaps reflecting that the pattern of selection into migration and Facebook usage based on unobserved characteristics shifted as a result of this policy change.

In 2022, we estimate that 25.6K people migrated from Ukraine to Sweden, far above the 800 migrants that the Swedish government reported to Eurostat. We believe that this distinction is driven by the fact that the Swedish government had not included most Ukrainians resettled under the Temporary Protection Directive in the population register used to calculate migration figures[17]. In 2022, the Swedish government separately reported that it had granted Temporary Protected Status to 47 thousand people after the Russian invasion of Ukraine[18], which supports a substantially higher estimate of migration from Ukraine. We do not add these recipients of Temporary Protected Status to our validation figures due to discrepancies in the migration concept considered. Concretely, not all those who are granted Temporary Protected Status meet the standard definition of migrants, as individuals counted in this enumeration may return home or move to a third country before spending a year in Sweden.

Germany. To further assess the quality of our data at the monthly level, we compare our data against data from the German government on entries at the monthly level. The German government issues these figures at a monthly level, which makes this an appealing source of high-frequency validation data. With that said, the definition of migration used in constructing this data differs from our own in several key ways, leading to a difference in the level of the two series. Concretely, the German data measures the number of arrivals from abroad who declare their intention to remain in Germany for more than three months, rather than the twelve months required by the United Nations definition of migration[19]. The German data also does not require that the migrant be present in their previous location for a year.

In Fig. S10, we plot the level of migration in the German administrative data against our estimate of the total level of migration to Germany. The two series have very similar patterns in terms of the temporal variation in the rate of migration, though the overall levels differ due to the looser definition of migration used in the German data. We also observe a slight lag in our data, which seems to lag the administrative figures by about one month, a pattern that does not appear in the New Zealand validation exercises presented in Fig. 4. This discrepancy could reflect a difference in administrative procedures or definitions across countries or a factor that affects the speed at which one's Facebook home prediction updates. This distinction is likely to be more muted at higher levels of temporal aggregation.

United States. U.S. Customs and Border Protection (CBP) shares the number of migrants encountered at the border each month [20]. In recent years, such encounters have accounted for a large share of migration to the United States from several countries, reflecting in part the spike in asylum claims initiated at the southern border, which normally involve migrants coming into contact with CBP agents. Many migrants whose presence is handled under the agency's Title 8 authority are allowed to stay in the country while their claims are pending, which often takes several years due to long backlogs in the immigration court system[21][22].

Comparing our estimates to the estimated number of individuals processed under Title 8 is difficult for a number of reasons. Importantly, the two measures define migrants' origins differently—we define migrants' origin using the country in which they last lived for 12 months, while CBP uses the migrant's citizenship. The figures presented by CBP also do not include non-asylum migration, such as students and people for work, which is present in our data. As a consequence, in certain cases our estimates of migrant flows diverge from those reported by CBP. For instance, these differences are strong in the case of

[¶¶¶] For more information on this policy, see here.

[17] For more information on this topic, see here. Some exceptions apply, such as migrants who are the partner of an EEA citizen.

[18] See Eurostat for these figures.

[19] Additionally, this definition is based on the intended duration of one's stay, not the true duration, as in our implementation.

[20] More information about this dataset can be found here.

[21] Data compiled by Syracuse University's indicated that the average wait time for a hearing in 2020 was 1552 days, over 4 years. Further statistics on processing times are available here.

[22] CBP also provides information on Title 42 encounters, which refers to provisions under which the government could process asylum claims in an accelerated fashion due for public health reasons. Most migrants processed under this provision do not remain in the United States for a year; as such, we do not compare encounters conducted under Title 42 authority to our data. This provision was widely used to turn back asylum seekers after the onset of the COVID-19 pandemic, before authorization for the provision was revoked in May 2023.

migrants from Haiti, where CBP reports a considerably larger flow than we measure in our data. In part, we believe that this reflects the fact that many Haitian citizens arriving in the United States actually left their home country after the 2010 earthquake and lived during the interim in Latin America[23]. Using our definition of migration, these Haitian citizens arriving in the United States are counted as migrants from the last country in which they spent one year.

Conversely, our estimates of migration from Mexico to the United States are higher than the number of encounters reported by CBP. In part, this reflects the high level of non-asylum migration from Mexico to the US, but our higher levels may also reflect the fact that many migrants from third countries spend a substantial amount of time in Mexico before arriving in the United States. In some cases, these delays can be substantial, as in 2019 when Mexico paused its issuance of exit permits for third-country migrants bound for the United States[24]. Migrants who remain in Mexico for more than a year as a part of their journey to the United States are classified as migrants from Mexico under our definition, but migrants from their country of citizenship under the definition used by CBP.

Eurostat. In Fig. S11, we present analogous validation exercises using the 2019 Eurostat estimates for a wide array of countries. Although Eurostat publishes the estimates in a standardized format, the collection procedures underlying this data vary by country. Some nations, such as Sweden, derive their estimates from high-quality population register data, while others use sample surveys, healthcare records, or a number of other techniques[25]. Despite this heterogeneity in the underlying quality of the data, we find that our data is in general highly correlated with the figures from Eurostat, with a correlation above 0.9 in 15 of the 21 countries reporting migration figures in 2019. We see similarly strong performances in Figures S12, S13, and S14, which repeat these validation exercises in 2020, 2021, and 2022.

With that said, we do find several large outliers in the data which could be informative to highlight. In the case of Bulgaria, we estimate that flows into the country from the United Kingdom and Germany greatly exceed the government estimates. We find a similar pattern in Romania, which sees higher inflows from Germany, the United Kingdom, and Italy than the government figures suggest. Although we do not have definitive evidence, it seems plausible that these flows represent Bulgarian and Romanian nationals returning to their native country after working or studying abroad. Both Bulgaria and Romania saw large-scale emigration to the higher-wage countries of Western Europe after labor mobility barriers were lifted in 2014, and have since seen individuals return in the wake of improving economic conditions locally and following the legal uncertainty for EU nationals imposed after Brexit. Official statistics may undercount such return migrants if they do not respond as other migrants do to questions about their migration status (23). We find a similar pattern in North Macedonia, where our estimates of the flow into the country from Germany are substantially larger than the level reported in the Eurostat data, which might reflect dual citizens or temporary workers returning from working in the country. The large flow we find to North Macedonia from Germany, which has both a strong labor market and a large stock of Macedonians, is consistent with this interpretation.

In some countries, we find that our estimates have a strong correlation with the figures from Eurostat, but the overall level of migration we detect differs substantially from the administrative figures. We find this most dramatic in the case of Slovakia, where our figures have a Pearson correlation of 0.33-0.97 across years, though our estimates find a substantially higher level of immigration than the government reports. We find that this discrepancy is likely caused by the method used by the Slovak government to report their figures to Eurostat—they include only individuals who have been granted a permanent residency permit[26]. The majority of migrants enter on temporary permits, which are renewable for several years. Such permits enable these temporary permit holders to meet the United Nations' definition of migration, which only requires migrants to reside in their destination country for one year[27]. These temporary residence permits are far more common than the permanent variety, with the OECD estimating that 20,000 temporary permits valid for more than one year were issued in 2019, relative to the 7,000 total migrants reported in the Eurostat figures that year[28]. For this reason, we think the estimates we report better capture the United Nations' definition of migration than the figures Slovakia provides to Eurostat.

In the case of Croatia, we find smaller levels of migration from Bosnia and Herzegovina (BA) and Serbia (RS) than are suggested by the administrative data. It is possible that our estimates here are complicated by people who hold dual citizenship across the countries, and who move between them relatively frequently, a problem raised by Dario Pavić et al. (24). It is possible that the people the official statistics record as moving to Croatia from these origin countries did not reside in their country of origin (or, perhaps, Croatia) for more than 12 months and thus do not meet our criteria for migration.

OECD. The Organisation for Economic Co-operation and Development (OECD) releases the International Migration Outlook, a comprehensive report that provides the number of permanent migrants to each OECD country, as well as in-depth analyses of current migration trends and policies[29]. The definition of permanent migrants in this report is irrespective of the actual duration of stay. Instead, it is based on the type of permit the individual uses to enter the destination country, except in cases of migration within free-circulation areas where no permit is required. This definition also includes individuals who transition from a temporary permit to a permanent one. Other definition differences exist; some countries, such as Australia, Ireland and the United States report their migration statistics using fiscal years instead of calendar years.

[23] See this article for a description of the process of migration that led such migrants to the United States.

[24] See here and here for more information on this policy shift.

[25] More information about the procedures used can be found here and here.

[26] More information about the Slovak government's definition of migration can be found here.

[27] Information about temporary residency permits in Slovakia can be found here.

[28] For more information about the temporary permits, see here.

[29] See International Migration Outlook 2022 by OECD for the details

More fundamentally, the OECD's definition of migration differs from our own in two key ways. First, their definition does not account for returned residents. Second, it does not include "temporary" migrants who live in a country for more than one year, such as students and workers. These factors can explain why our estimates are higher than OECD; the OECD estimates that 6.1 million people migrated to OECD countries in 2019, while our estimate stands at 12.1 million (Fig. S15). For example, the NSO of New Zealand reported that the nation received 165,741 immigrants in 2019 (6), while the OECD reported that there were only 38.3K immigrants to New Zealand in that year[30]. Overall, our estimates exhibit a strong positive correlation with the OECD's figures (Pearson correlations of 0.87 in 2019, 0.82 in 2020, 0.81 in 2021, and 0.92 in 2022) despite the differing migration concepts employed in the two datasets.

Global indirect estimates. To incorporate a more global perspective for our validation we compare our estimates against indirect estimates of global migration. In recent years, the United Nations and World Bank have both published data on migrant stocks, broken down primarily by their country of birth in five-year intervals. Several methods have been developed to indirectly estimate the migration flows to match the changes in migrant stocks over the five-year intervals (8, 25). These methodologies estimate migrant transitions, that is, individuals who resided in country i at the start of the five-year time period and in country j at the end of the time period, regardless of any other moves made in the interim. This transition-based definition of migration undercounts the total number of moves during the five-year time period as return moves within the interval are not captured in the changes in the migrant population data.

The use of indirect migration estimates as a validation source also presents some challenges. Most notably, the stock figures on which the estimates are based are only available every five years, so we are forced to compare our migration estimates for a single year (2019) to the average annual rate of migration over the 2015-2020 period in the indirect estimates. The definition of migration also differs across the two concepts, as the indirect estimates employ a transition-based, rather than a movement-based, definition of migration. For this reason, we find that our correlations with this source of validation data are lower than our correlations against alternative validation sets (see Fig. S16). Nevertheless, we find our estimates correlate best with indirect estimates from the Pseudo-Bayesian approach within the demographic accounting closed system (26), which themselves have been shown to provide the closest match to reported migration figures of the six methods considered (8).

Alternative validations of migration. In Tables S5, S6, S7, and S8, we present validations using the data sources above on a number of alternative migration measures. In these exercises, we transform both our data and the administrative data before correlating the two series. For each metric, we present the number of observations included in the comparison[31]. When calculating the aggregations, we compute totals solely over the set of country pairs included in both data sets. For instance, though we can observe outbound migration between almost all country pairs, not all countries who participate in the Eurostat dataset report their outbound migration to non-Eurostat countries. As a result, when we calculate the aggregate level of migration from these countries in the Facebook data, we exclude migration that involves a country pair not observed in Eurostat.

In general, our data is strongly correlated with the administrative data across the different measures that we examine. There is a generally lower level of correlation across data sources after we apply the log transformation. This is largely due to the effects of our differential privacy protections, which add relatively large amounts of noise to country pairs with small estimated migration flows. This noise has a larger impact on the overall correlation following the log transformation.

Estimates for China

In most of this paper, our migration estimates cover the sample of 181 countries defined in Table S9. These countries account for around 79% of the world's population. China accounts for the bulk of the remainder of the population, but the low level of Facebook usage in the country makes it difficult to estimate migration involving the nation with our standard methodology. With that said, there are a substantial number of Facebook users in China, despite the low penetration rate. In this section, we use an alternate methodology to estimate the level of migration to and from China at the population level.

To do this, we make use of data from Eurostat, which reports migration from China to 21 countries in 2019. We fit a simple linear model without an intercept:

$$\text{MigEurostat}_{\text{China},d,2019} = \beta \text{MigFB}_{\text{China},d,2019}$$

Here, $\text{MigEurostat}_{\text{China},d,2019}$ is Eurostat's figure for migration from China to country d in 2019 and $\text{MigFB}_{\text{China},d,2019}$ is the number of users in our sample who migrate to country d from China in that year. We find that $\beta = 11.36$ is the best fit and use this factor to rescale all of our raw estimates of migration to and from China. In Fig. S17, we show that the resulting estimates of migration from China have a very high correlation with the estimates from Eurostat[32].

Using this rescaling technique, we estimate that 2.04 million people migrated from China to another country in 2019[33]. We show in Fig. S18 that migration fell dramatically following the onset of COVID-19 before recovering during 2021.

[30] See Table 1.1 in the International Migration Outlook 2023 by OECD

[31] This varies across rows for several reasons. In the row correlating the log of each series, we must exclude country pairs in which either of the series records no migrants, as the log of 0 is undefined. For the total outbound, total inbound, and net migration rows, we aggregate the country pair level to the country level.

[32] Migration from China to countries outside of Europe might be driven by a different pattern of selection. Unfortunately, we do not know of any validation data that would allow us to benchmark the performance of our technique in the developing world.

[33] As we do not know the nationality of Facebook users, it is important to note that our estimated number of migrants from China should not be assumed to represent Chinese nationals who have relocated to another country.

National income levels and migration trends

The global scale of our dataset allows us to explore how international migration patterns vary with the development level of the origin and the destination. We find that migrants tend to move to relatively wealthy countries: countries classified as high-income by the World Bank attract 67% of the global migrants. Correspondingly, only 6% of global migrants choose low-income countries as their destinations.

High-income countries are also over-represented as sources of migrants. High-income countries comprise just 19% of the world population but represent 33% of global migrants. This pattern is driven by the combination of several factors. Globally, migrants from high-income countries are more able to finance migration and tend to enjoy a more favorable treatment in their destination countries. We explore this pattern in Fig. S19. In Europe a majority of migrants come from high-income countries, probably tied to the freedom of movement in the European Union. South Asia also sees large-scale inflows from high-income countries, which is driven by individuals originally from the region returning from labor contracts in Western Asia.

Crisis-induced migration

Our estimates can also help to illustrate the effect of conflicts and civil unrest on migration, in a context in which gathering more traditional indicators of migration may be difficult due to logistical or political sensitivities. In Panel A of Fig. S20, we highlight the particularly large outflows from Ukraine following Russia's invasion of the country in February 2022. In total, we estimate that 2.3 million people emigrated from the country and settled elsewhere for at least a year between February and December 2022, a tenfold increase over the pre-war emigration rate. In Panel B of Fig. S20, we show the top five countries that received the most migrants population – Poland, Germany, Czech Republic, the United States, and United Kingdom[34]. Our estimates are lower than the 3.8 million recipients of Temporary Protected Status (TPS) reported by Eurostat in January 2023[35], which we believe is due to our more conservative concept of migration. Concretely, the Eurostat figures do not impose a minimum length of stay in either the origin or the destination—for instance, a migrant who moved to Poland in October 2022 and applied for TPS before returning to Ukraine in August 2023 would be counted in the TPS figures but not in our migration estimates. Most migrants from Ukraine in 2022 planned to return to the country, with a survey by the International Organization on Migration finding that 77% intended to return[36].

Fig. S21 shows the top 20 destination countries from Ukraine during the Ukraine war in terms of the total number of migrants (Panel A) and the proportion of migrants over the population in the destination country (Panel B). Different from the rank based on the total number of migrants, Lithuania and Estonia have a much higher proportion of migrants over the population – the migrants from Ukraine between Feb. 2022 to Dec. 2022 account for 1.14% and 1.97% of the population in Lithuania and Estonia, respectively.

In Fig. S22, we demonstrate the rapid increase in outward migration flows from Myanmar following the February 2021 coup. Disaggregating these migrants by destination, we observe particularly large increases in India and Thailand. We notice a similar outward migration flow from Hong Kong following the passing of the controversial national security law in June 2020[37]. Most strikingly, outflows from Hong Kong to the United Kingdom increased fifteenfold in the four months following the passage of the law. Many of the departures preceded the United Kingdom's announcement of the British National (Overseas) visa program for Hong Kong residents in January 2021.

Social networks and migration

The role of social networks in predicting and facilitating migration has been of immense interest to economists, sociologists, and human geographers (14, 27–30), but has not previously been tested on a global scale. In this section, we produce figures connecting the aggregated migration flows with an index of social connectedness between countries. To measure migration flows, we calculate the migration intensity, which we define as:

$$Migration\ intensity_{A,B} = \frac{M_{A \to B} + M_{B \to A}}{Pop_A * Pop_B}$$

The Social Connectedness Index, which was introduced in (31), is defined analogously as: $SCI_{A,B} \propto \frac{Friendships_{A,B}}{FBUsers_A \times FBUsers_B}$

In Fig. S23, we present the correlation between these indices on the country pair by year level, using our index of migration to measure flows. We include only those country pairs with at least 20 migrants because 95% of the noise from differential privacy is in [-6.98, 6.98], which could cause outliers if the number of migrants is very small. We find that the two indices are highly correlated, measuring 0.75 in the full sample in 2022, rising to 0.90 when we limit the sample to only migration between OECD countries.

[34] Our measures of the top destination countries are closely aligned with estimates from the UNHCR; see here for more details.

[35] This figure is derived from the migr_asytpsm series.

[36] A summary of the survey's findings can be found here.

[37] See here for the details.

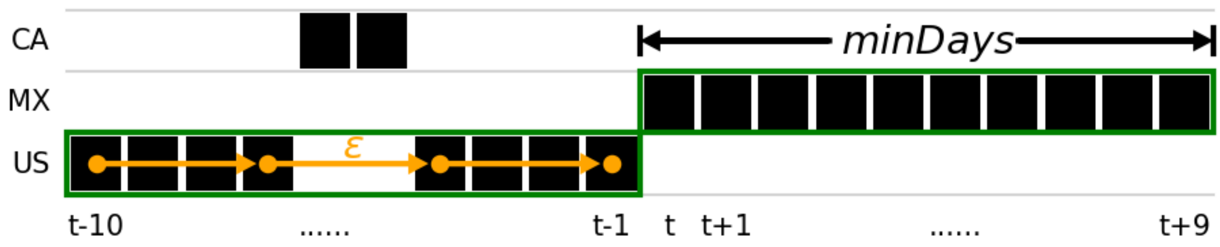

Fig. S1. Diagram of the segment-based algorithm, portraying the location history of one individual. Each row is one country, while each column is one day. The black square means this person lives in that country on that day. The green rectangles are two segments. ϵ defines the maximum gap between consecutive days. $minDays$ defines the length of each segment. $propDays$ defines the proportion of days in each segment. In this case, it is 80% in the segment of Mexico, and 100% in the segment of the US.

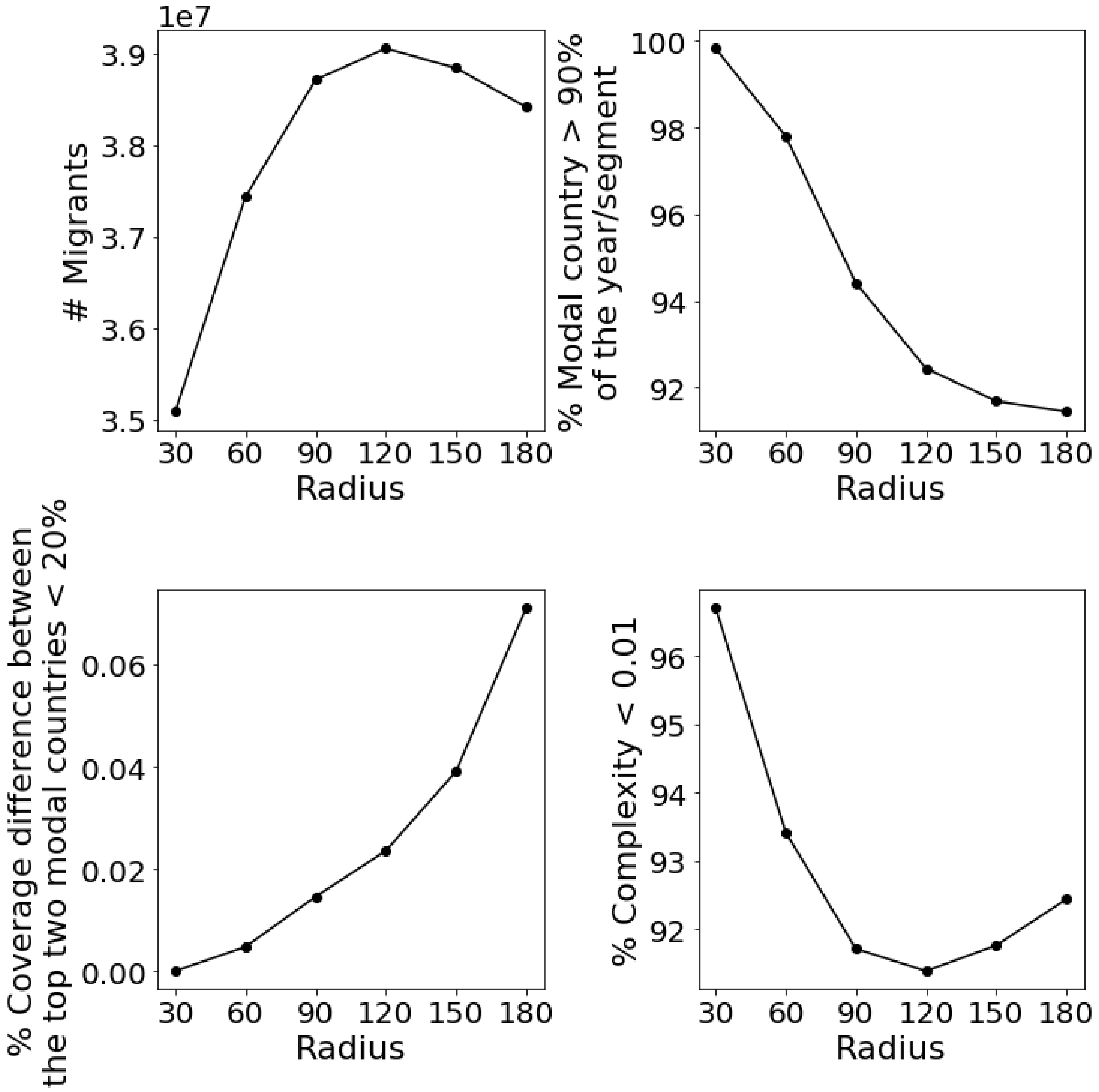

Fig. S2. Effect of different radii on migration results. The first plot shows the number of migrants detected using different radii. The three other plots show the effect of different radii on different metrics.

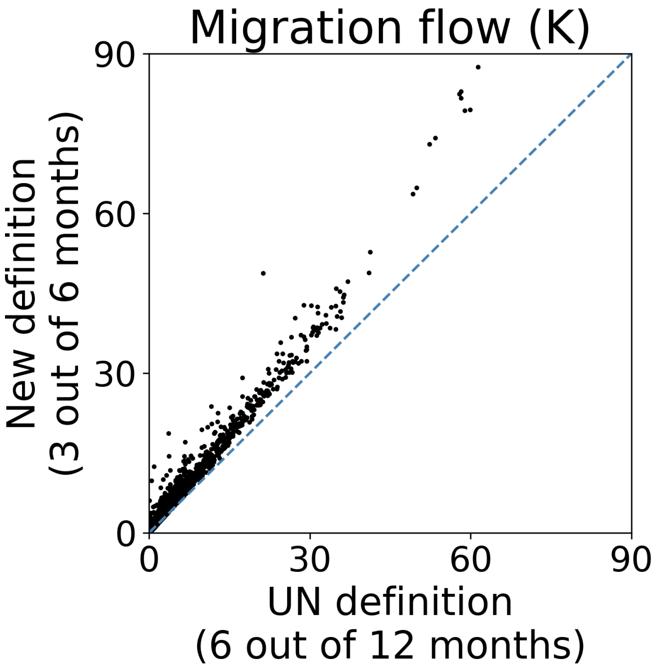

Fig. S3. Relationship between the number of migration flows based on the United Nation's recommendation (which requires residence in the destination for at least 6 out of 12 months) and based on the new definition (which requires residence in the destination for at least 3 out of 6 months), for each country pair and month in 2019.

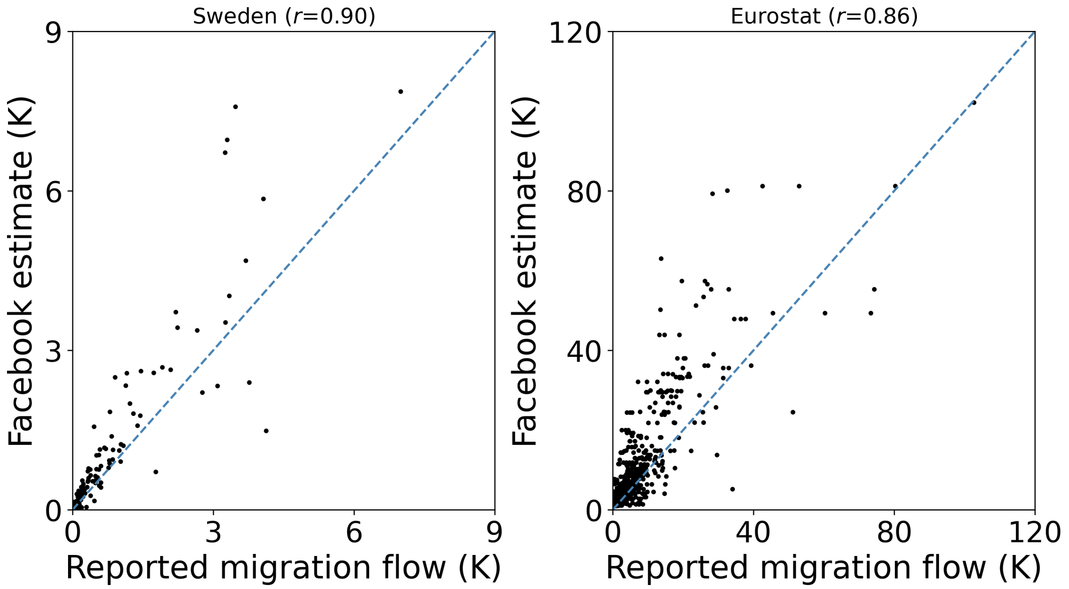

Fig. S4. Validation with Sweden and Eurostat migration datasets based on the new definition (a person living in a new place for at least 3 out of 6 months).

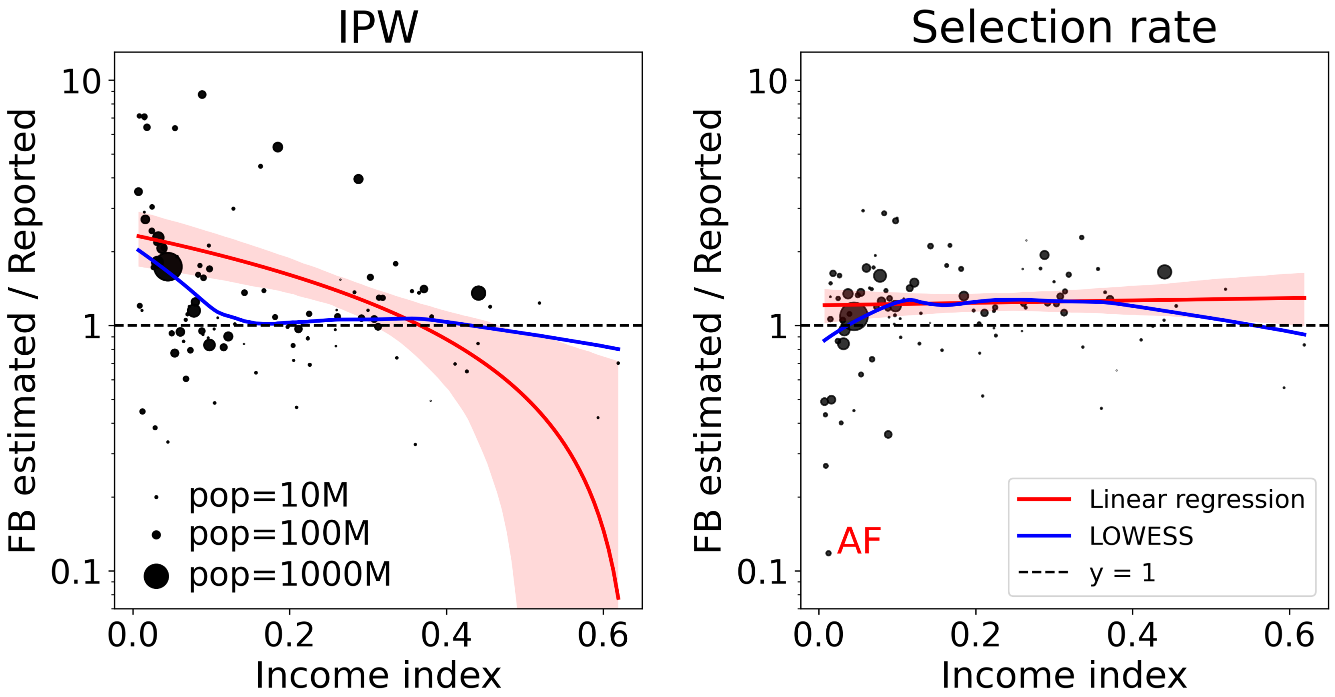

Fig. S5. Ratio of estimated flows to administrative records in 2019, using the inverse penetration rate weights (left panel) and the selection rate weights (right panel). The income index is described in Equation 2. Countries with under 100 migrants in the administrative data in 2019 are excluded from the plot.

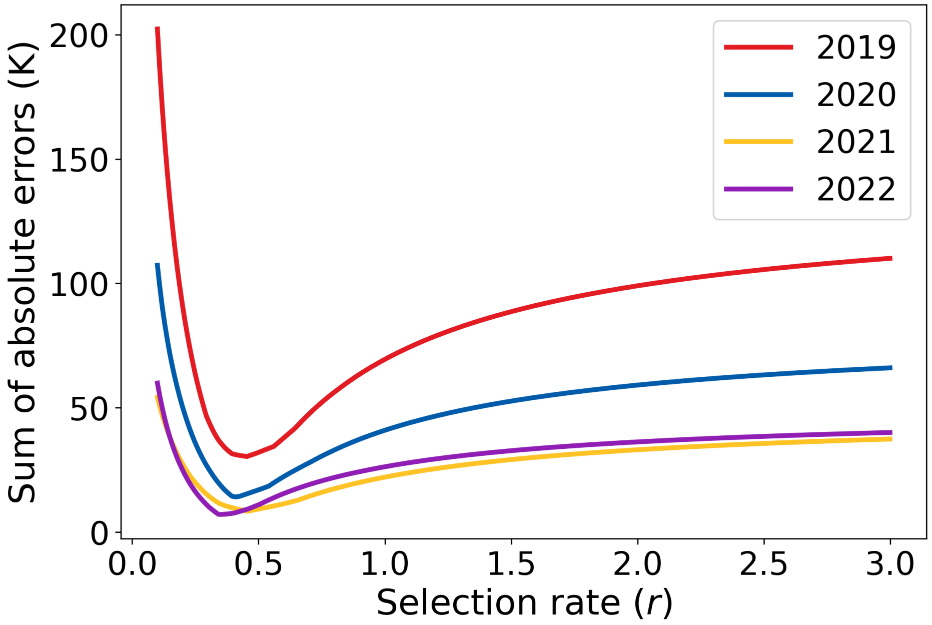

Fig. S6. The effect of selection rates on the sum of absolute errors based on the NSO dataset in Sweden. We exclude migrants from Ukraine to Sweden from these analyses since Sweden did not include those protected under the Temporary Protection Directive from its immigration figures; see SI Validation Sweden for more details.

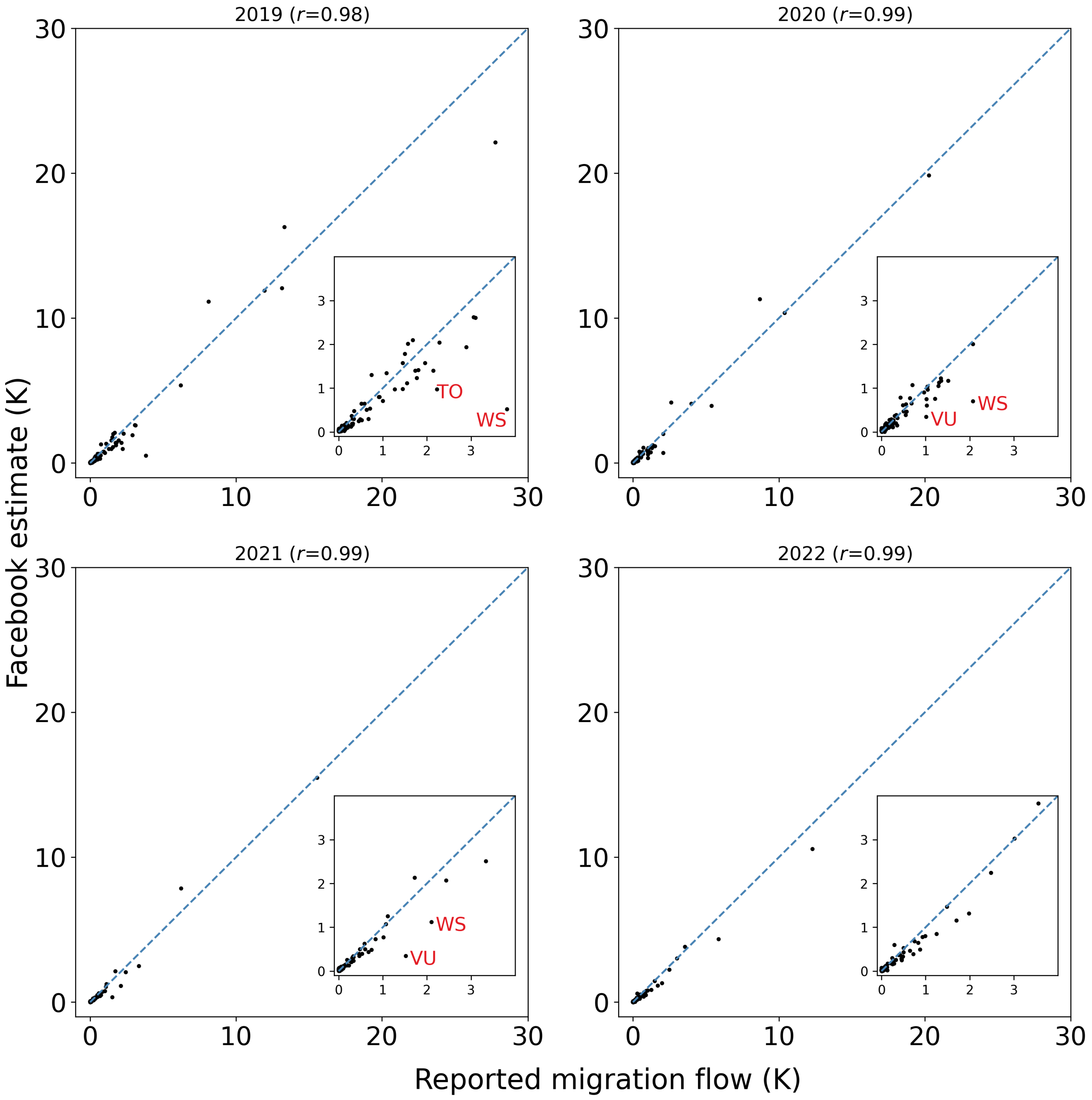

Fig. S7. Validation in New Zealand from 2019 to 2022. Each dot represents annual migration from an origin country to New Zealand. All axes are in thousands of people.

 Guanghua Chi, Guy Abel, Drew Johnston, Eugenia Giraudy and Mike Bailey

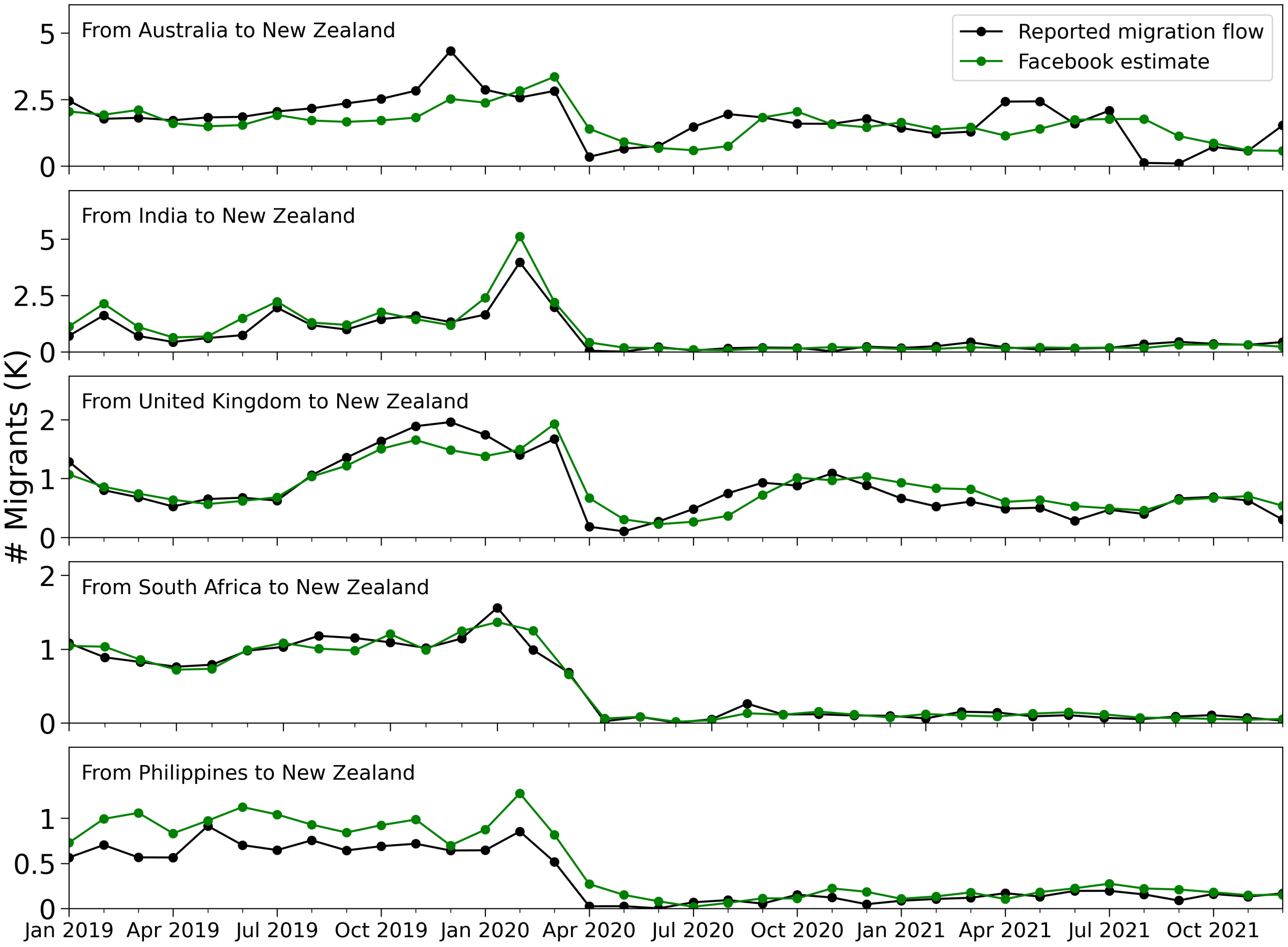

Fig. S8. Validation in New Zealand at the monthly level in the top 5 countries: Australia, India, United Kingdom, South Africa, and the Philippines.

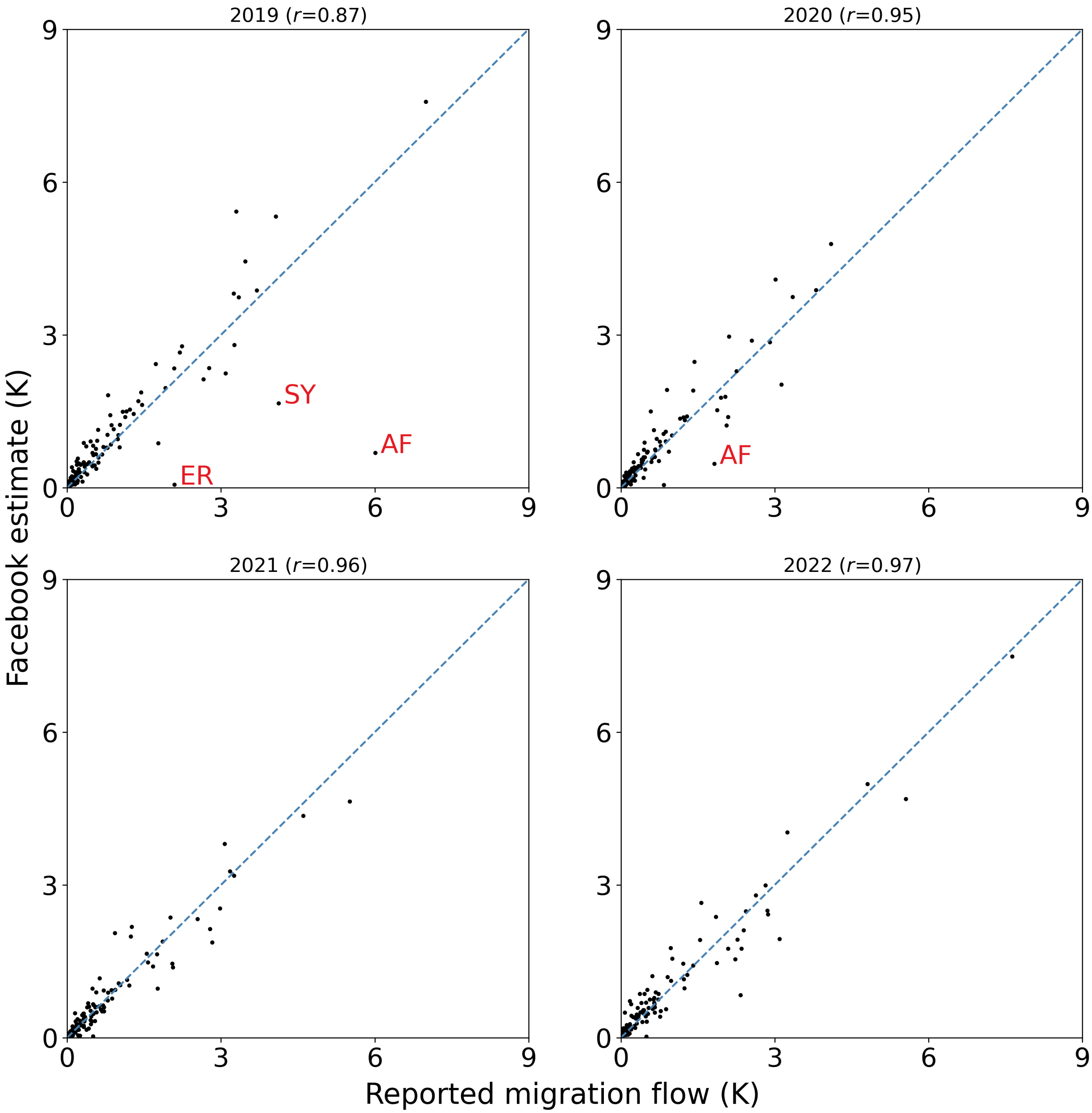

Fig. S9. Validation in Sweden in 2019, 2020, 2021, and 2022. Note that we do not add the point from Ukraine to Sweden in 2022 because our estimate of 25.6K is much larger than the scale in the y axis. The correlation in 2022 is calculated only using the data points shown in this plot (without Ukraine).

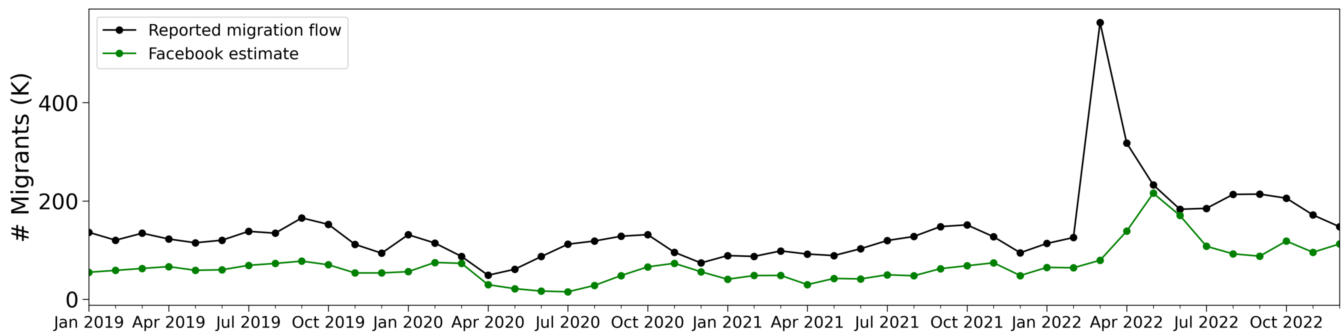

Fig. S10. Validation in Germany at the monthly level from 2019 to 2022.

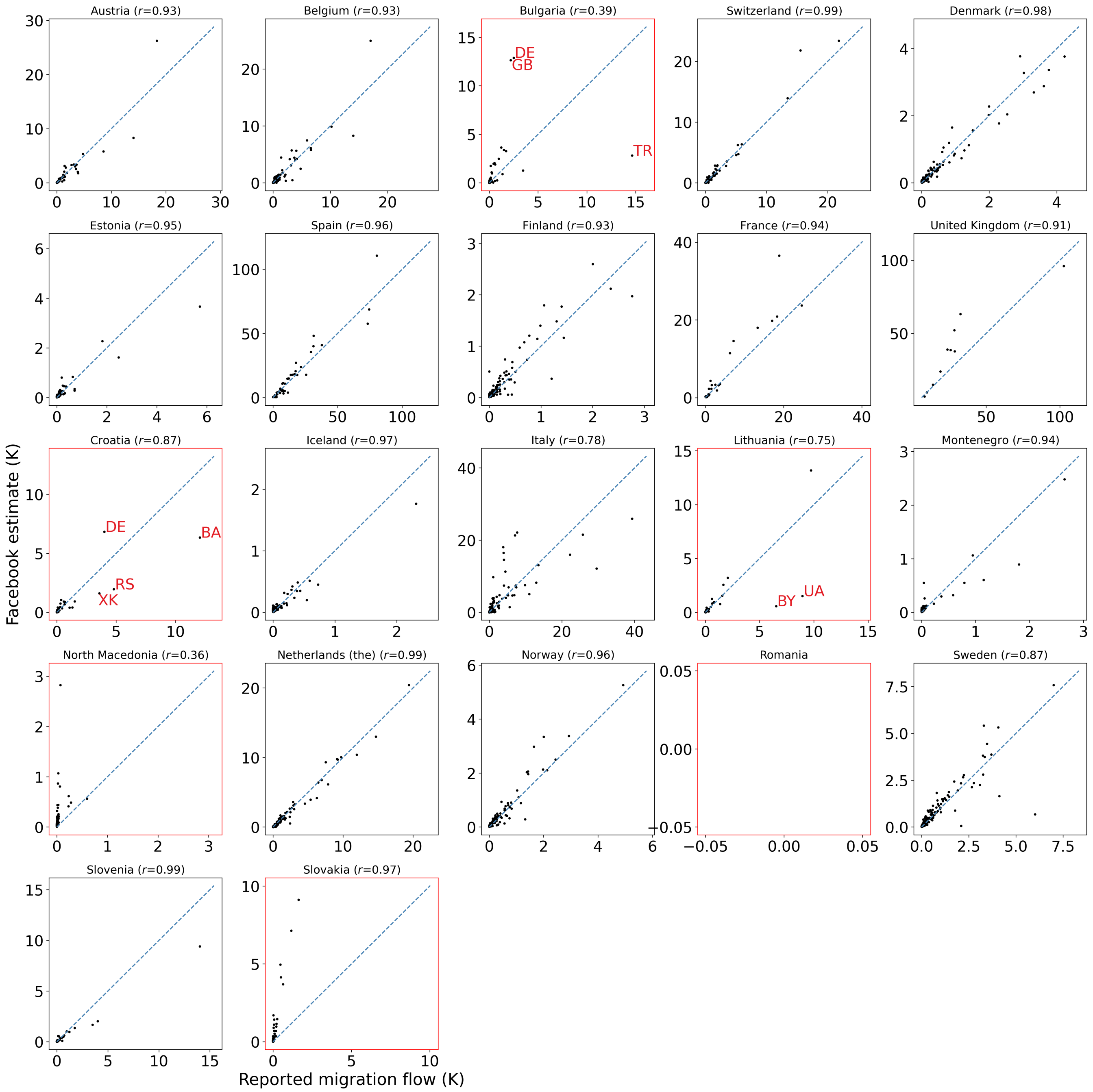

Fig. S11. Comparison of our estimated immigration figures for 2019 with those compiled by Eurostat. Each destination country is presented in a separate plot.

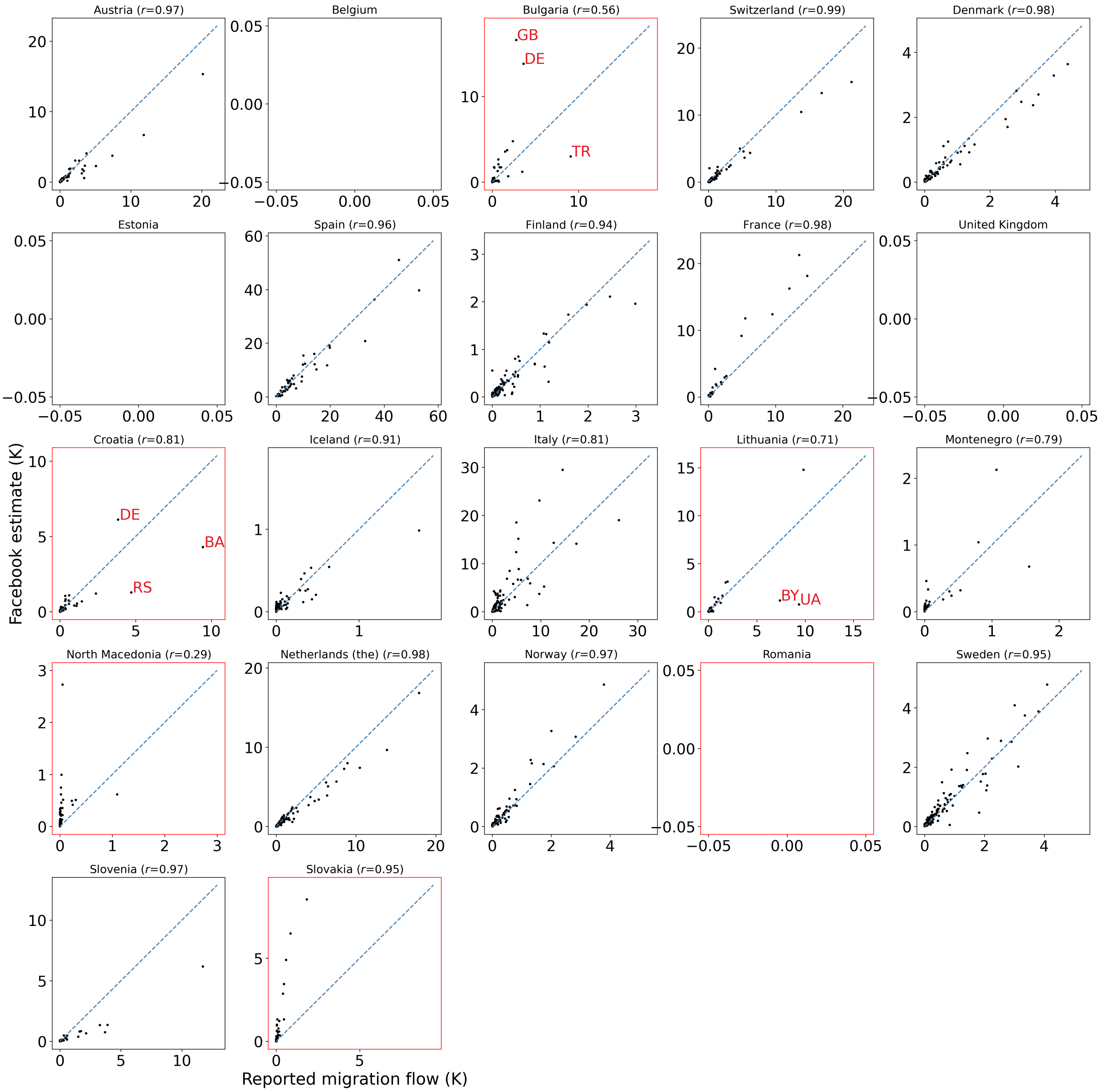

Fig. S12. Comparison of our estimated immigration figures for 2020 with those compiled by Eurostat. Each destination country is presented in a separate plot.

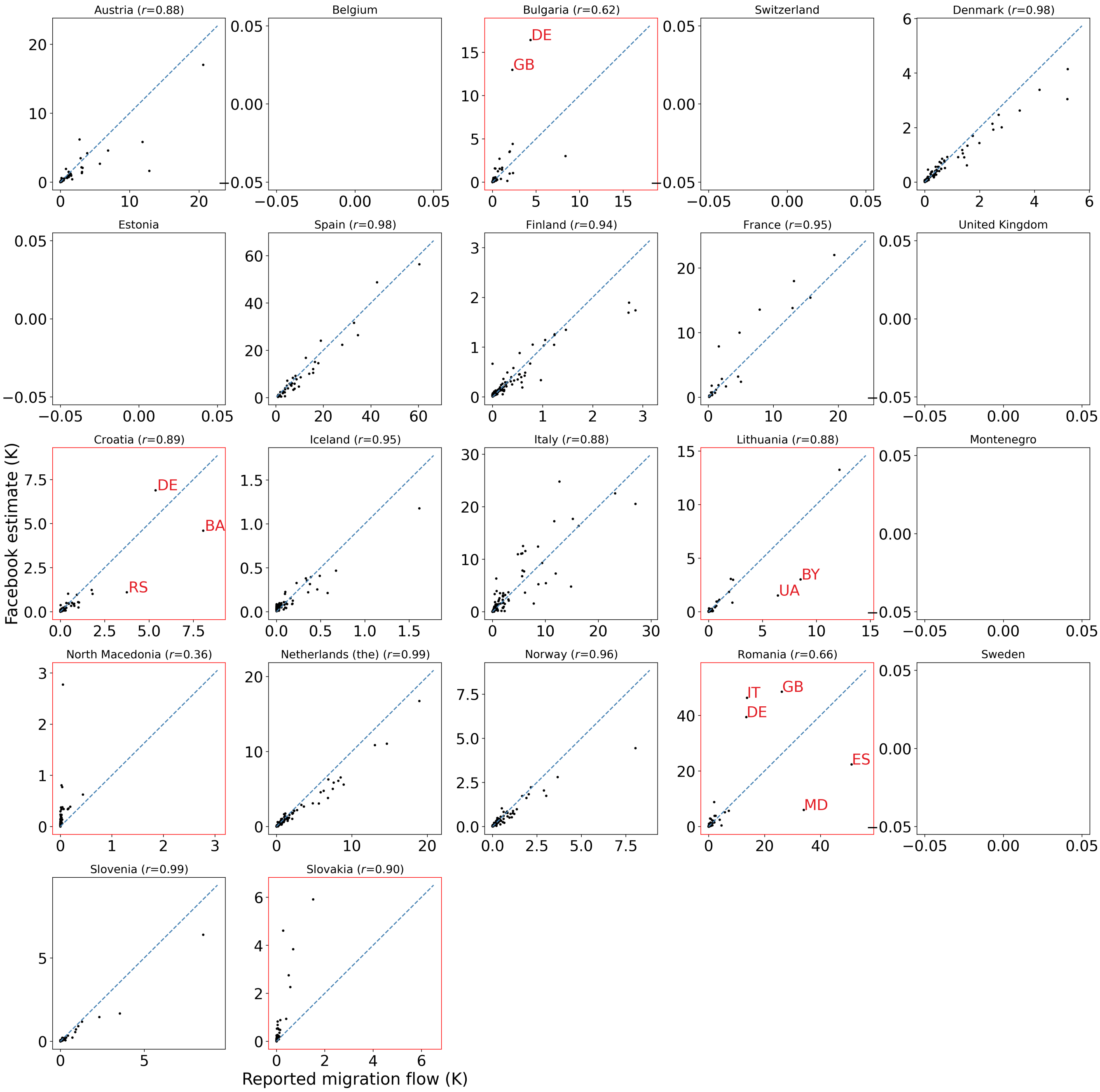

Fig. S13. Comparison of our estimated immigration figures for 2021 with those compiled by Eurostat. Each destination country is presented in a separate plot.

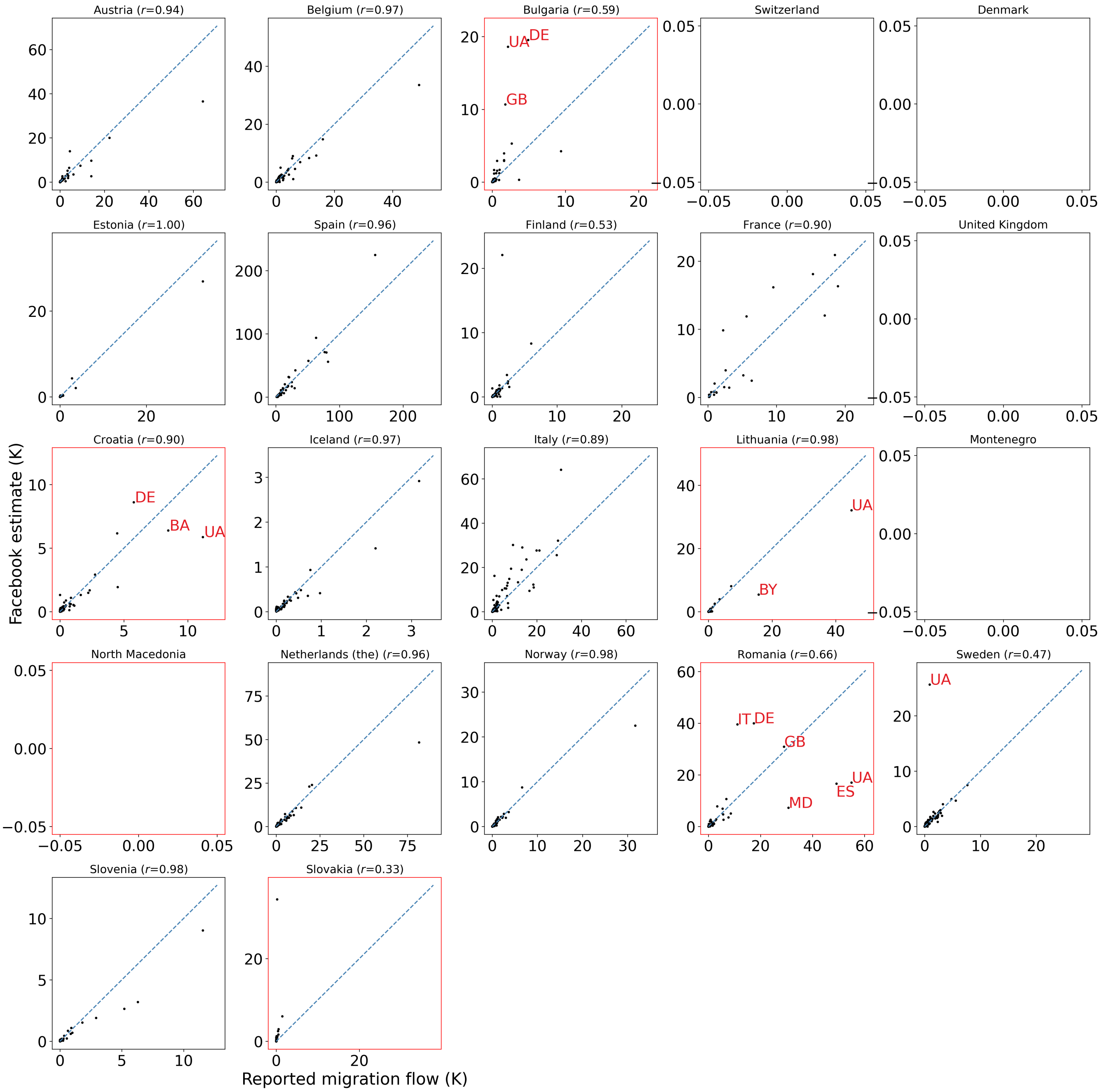

Fig. S14. Comparison of our estimated immigration figures for 2022 with those compiled by Eurostat. Each destination country is presented in a separate plot.

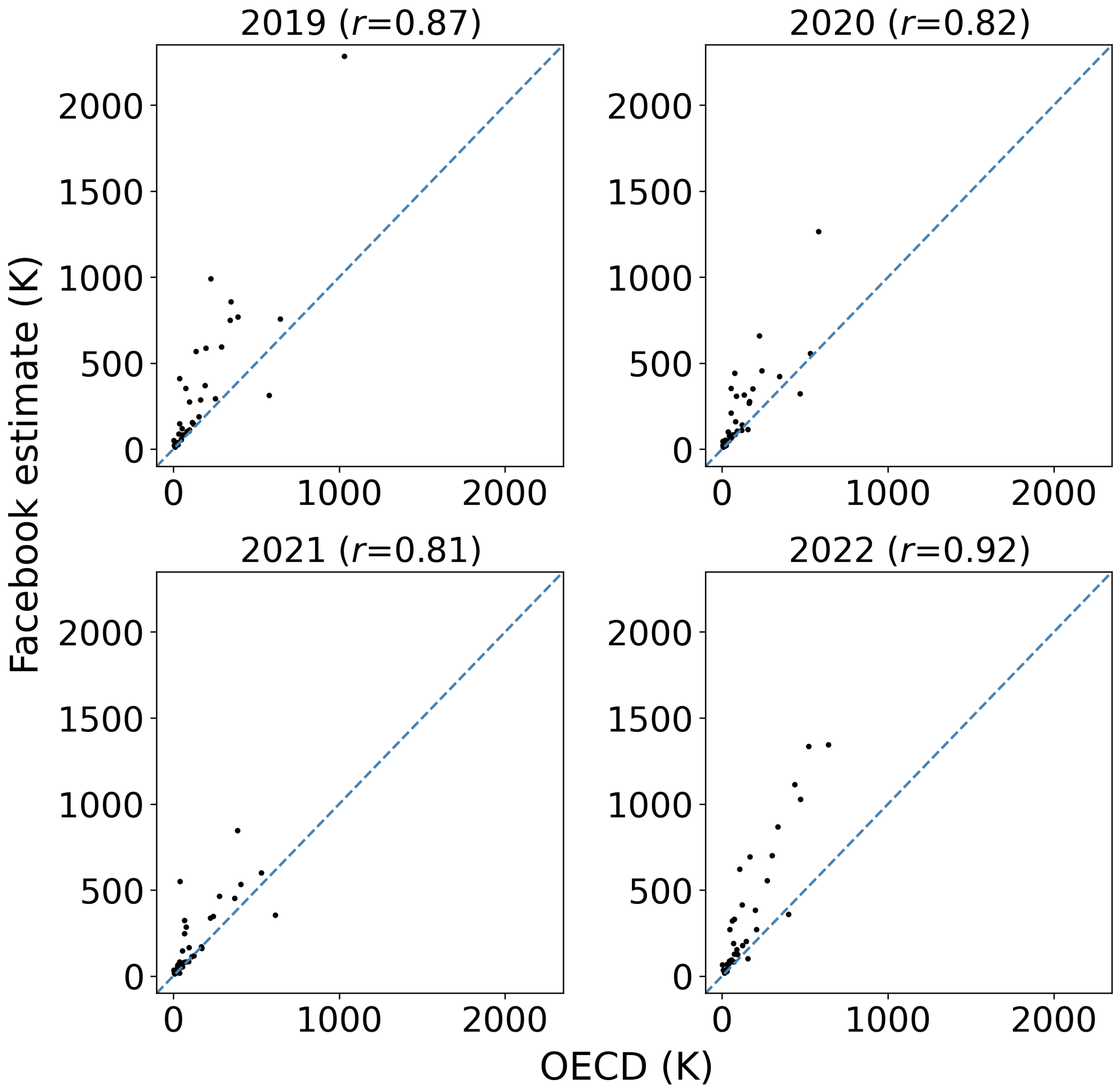

Fig. S15. Validation of in-flows in OECD countries.

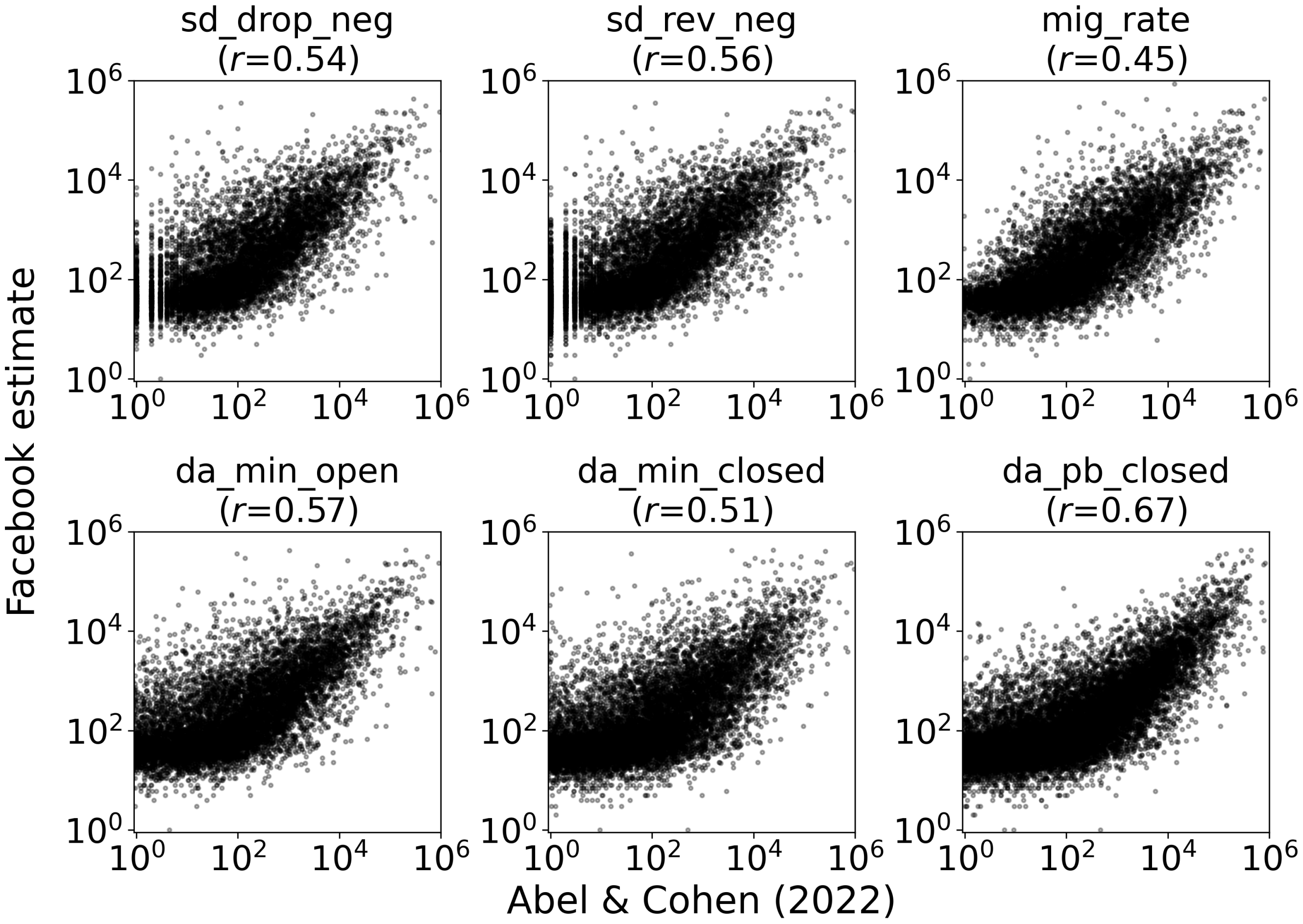

Fig. S16. Validation of country-to-country estimates flows during 2019 with updated estimates of (8) for the 2015-2020 period. Six methods were used in this paper, including (1) stock differencing drop zeros (sd_drop_neg), (2) stock differencing reverse negative flows (sd_rev_neg), (3) migration rates (mig_rate), (4) demographic accounting in an open system with a minimisation approach (da_min_open), (5) demographic accounting using a closed system with a minimisation approach (da_min_closed) and (6) demographic accounting using a closed system and a Pseudo-Bayesian approach (da_pb_closed).

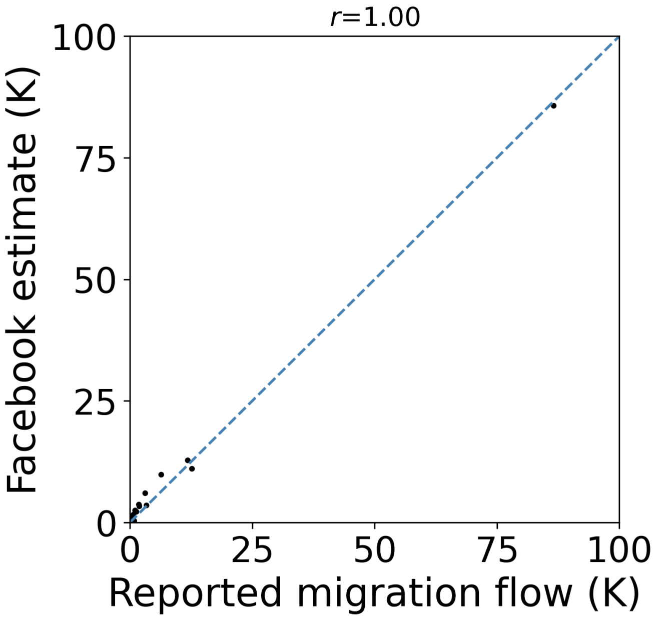

Fig. S17. Validation of our 2019 estimates of Chinese migration to 21 countries using Eurostat data

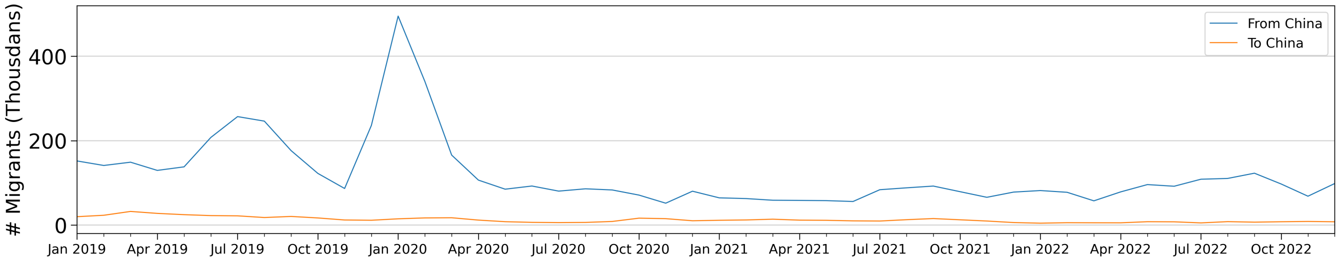

Fig. S18. Migration to and from China

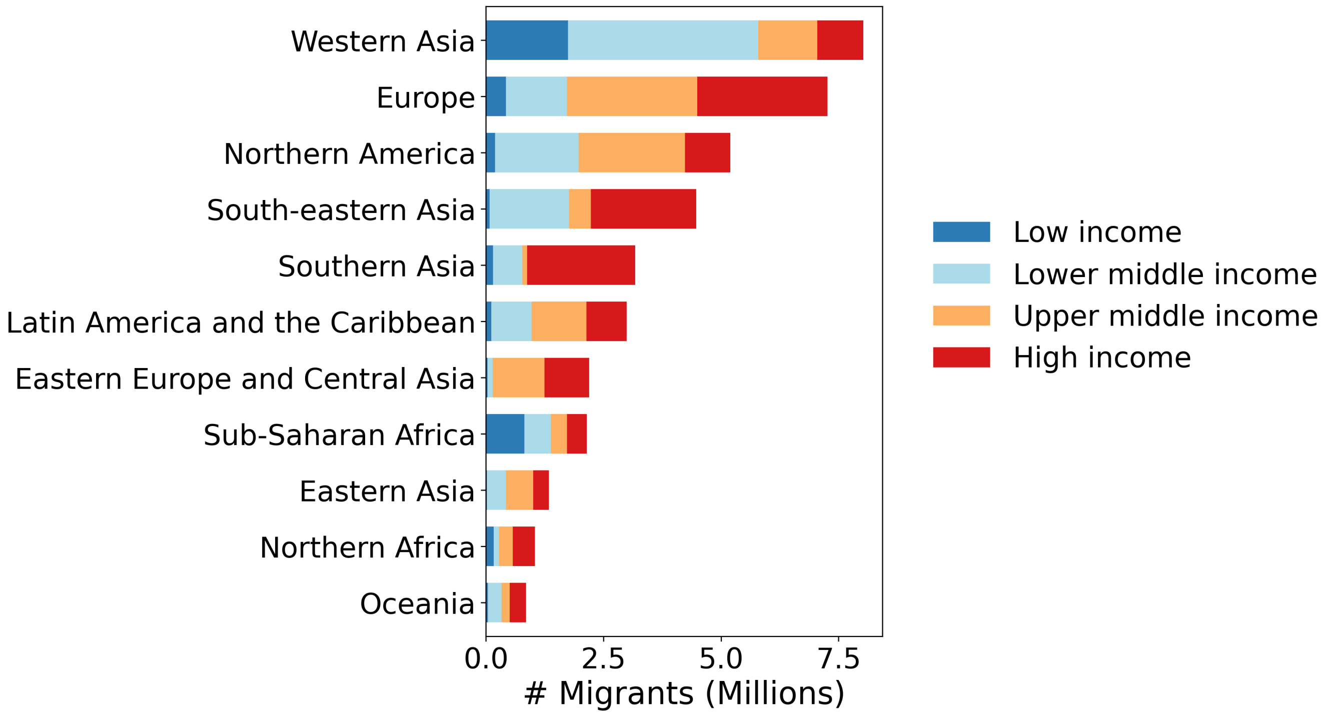

Fig. S19. 2022 regional migration by income level of origin country.

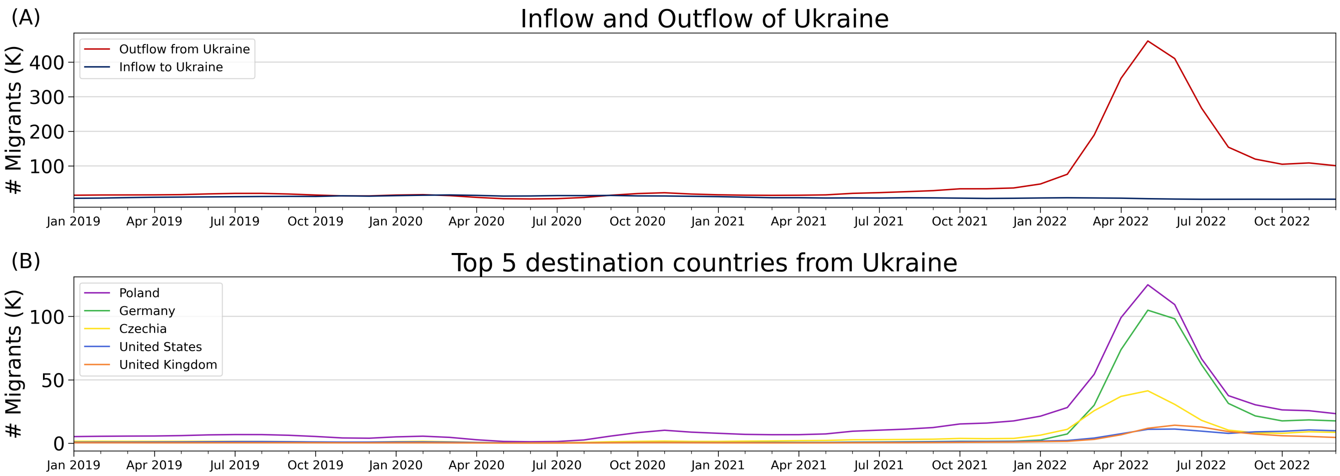

Fig. S20. Migration flow changes before and after the Ukraine war. Top: Inflow to Ukraine and Outflow from Ukraine from 2019 to 2022; Bottom: Top 5 outflow destinations from Ukraine during the Ukraine war from Feb. 2022 to Dec. 2022.

(A)

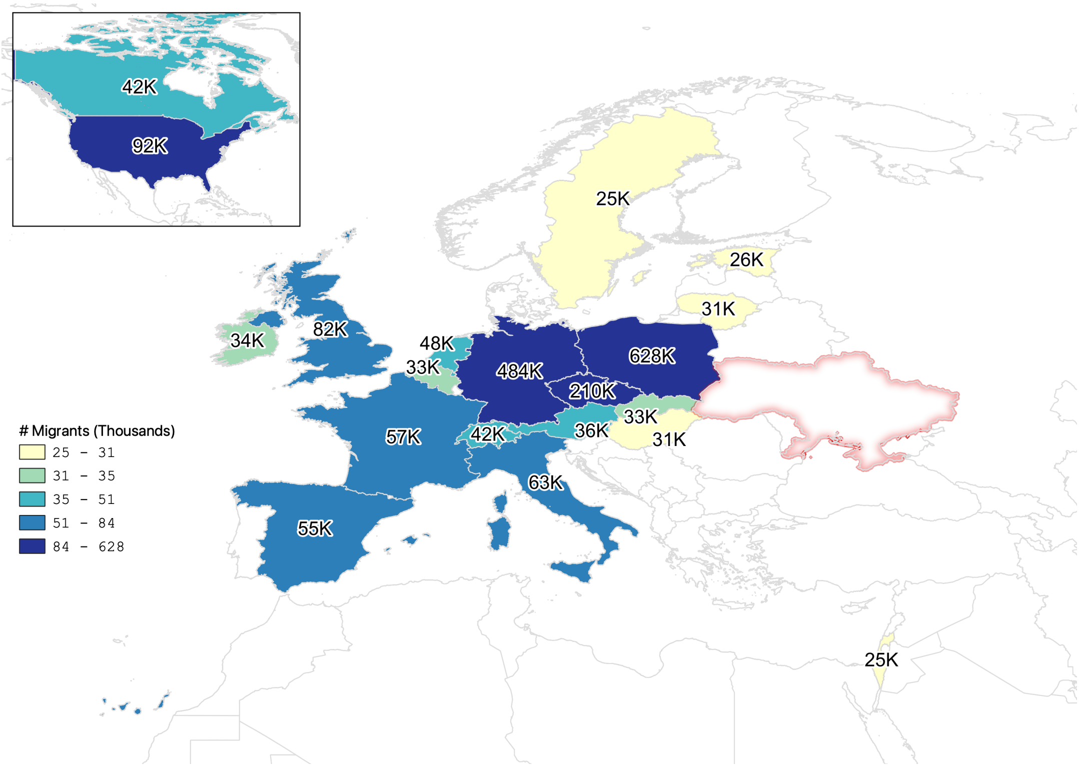

(B)

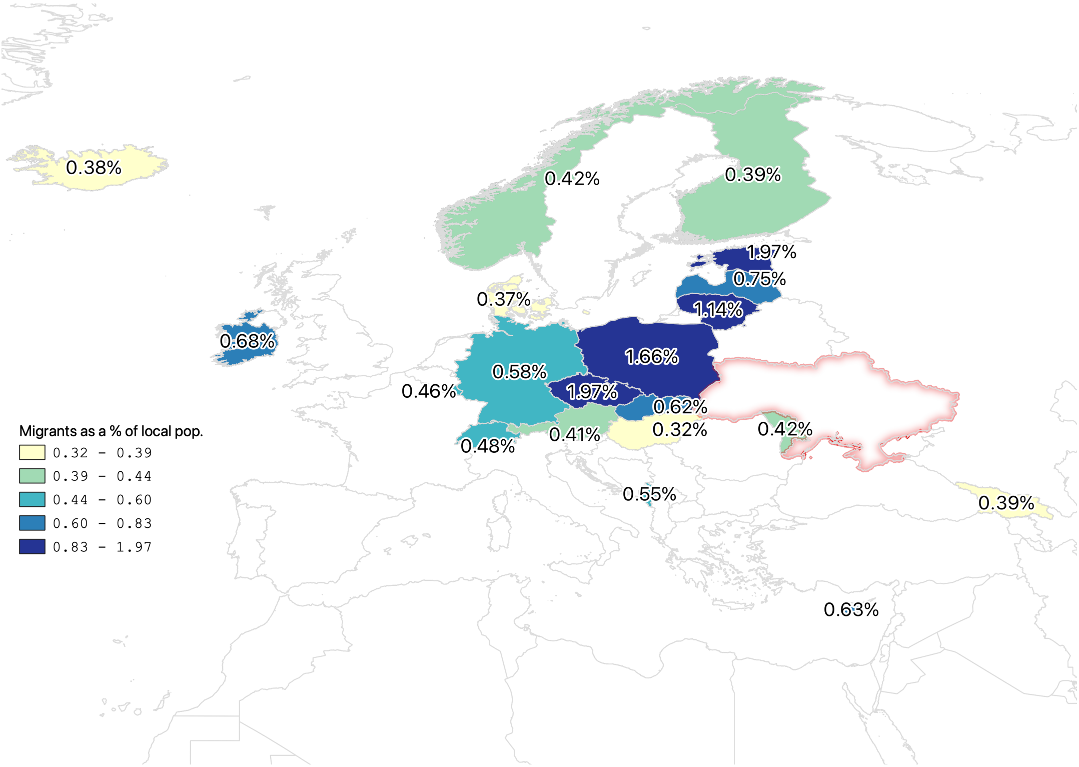

Fig. S21. Top 20 outflow destinations from Ukraine during the Ukraine war. (A) Total number of migrants from Ukraine in each country; (B) Proportion of migrants from Ukraine over the population in each destination country.

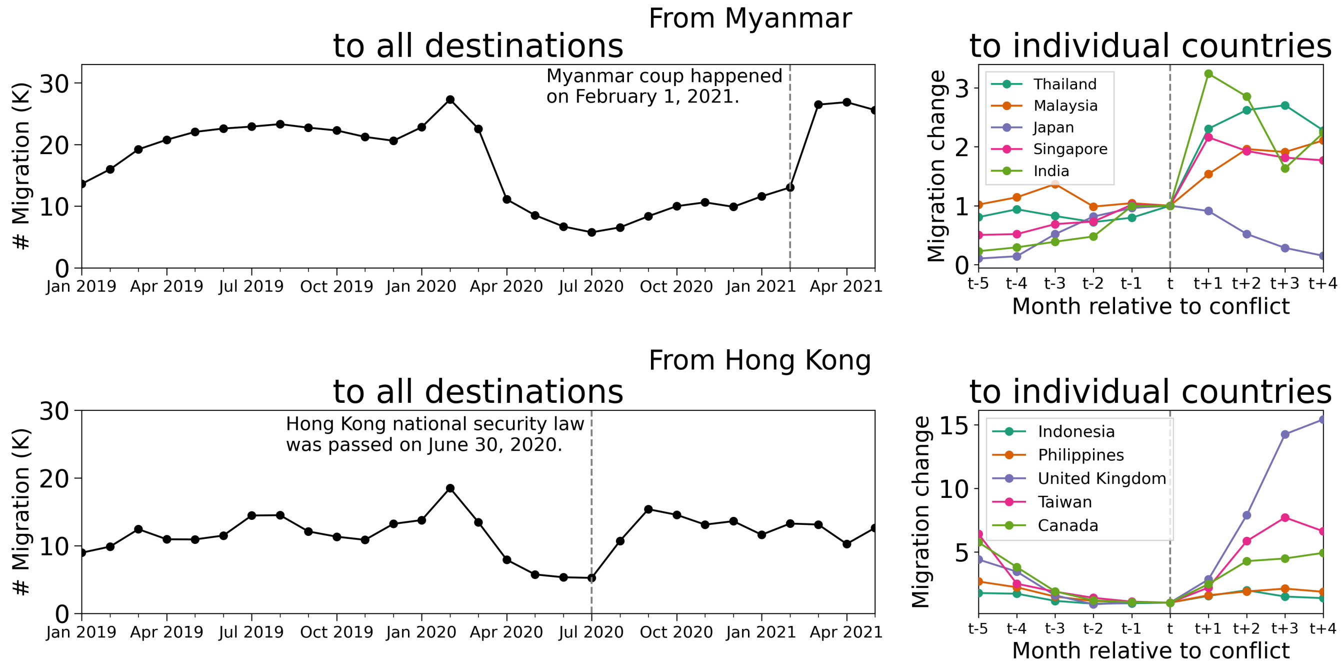

Fig. S22. Migration flow changes after crisis. Top: Myanmar coup in February 2021; Bottom: Hong Kong national security law in June 2020. The plots in the right column show the migration flow change relative to the month (t) when the crisis happened. The Y-axis is normalized so the value in month t is 1 for all destinations. The five countries are the top 5 destinations based on the number of migrants in our dataset.

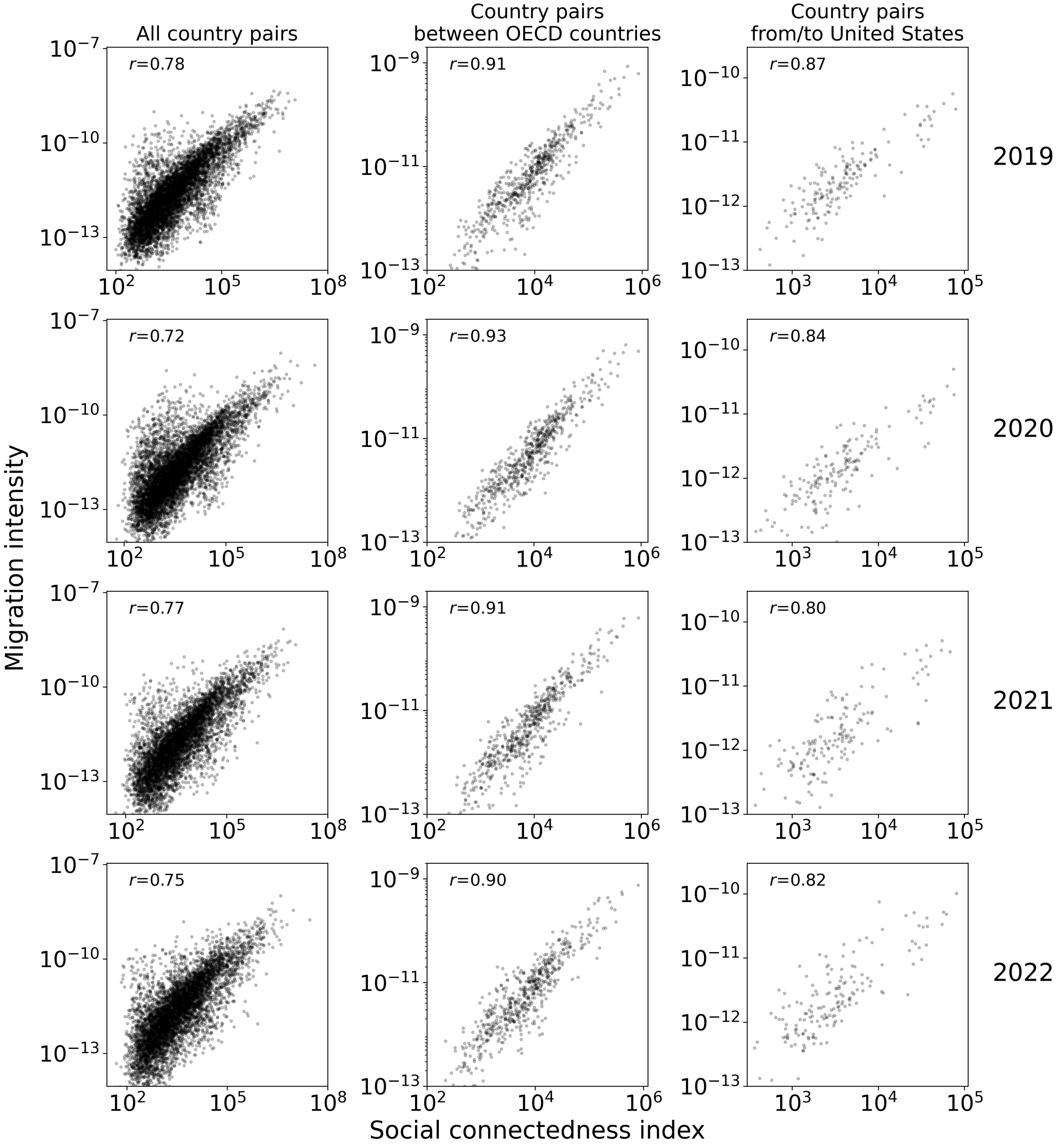

Fig. S23. Correlation between social connectedness index and migration. The first row is the correlation in 2019; the second row is the correlation in 2020; the third row is the correlation in 2021, and the bottom row is the correlation in 2022. The left column includes all the country pairs, the middle column only includes country pairs between OECD countries, and the right column only includes country pairs that include the United States as the origin or destination.

Table S1. Performance comparison of the segment-based and frequency-based methods

	Frequency-based (%)	Segment-based (%)
**Modal country**		
Share of users whose modal country covers $\geq 90\%$ of the year/segment.	70.5	97.8
Share of users for whom the difference between the most-common and second most-common countries is smaller than 20%.	6.6	0.004
Longest segment >300 days	81.3	100
**Complexity**		
Complexity <0.01	65.7	93.5

Table S2. Comparison of different weighting methods using data on migrants to Sweden. Errors are expressed in thousands of people. The High HDI and Low HDI rows subset to source countries that are, respectively, above and below the global median Human Development Index. In 2022, we exclude migrants from Ukraine from the analysis since Sweden did not include those protected under the Temporary Protection Directive from its immigration figures; see SI Validation Sweden for more details.

Year	HDI type	Raw		Penetration		Raking		Coefficient		Selection rate	
		r	error	r	error	r	error	r	error	r	error
2019	All	0.84	55.5	0.59	83.5	0.79	62.3	0.84	42.0	0.86	**39.0**
	High HDI	0.92	29.1	0.56	48.3	0.75	27.4	0.92	23.8	0.93	20.7
	Low HDI	0.80	26.4	0.80	35.3	0.82	34.9	0.80	18.2	0.80	18.4
2020	All	0.93	39.9	0.62	50.0	0.59	71.6	0.93	**22.2**	0.94	23.7
	High HDI	0.94	25.9	0.58	28.8	0.51	45.1	0.94	14.0	0.95	15.7
	Low HDI	0.92	14.0	0.76	21.2	0.84	26.5	0.92	8.2	0.93	7.9
2021	All	0.95	44.8	0.65	49.8	0.71	51.3	0.95	23.5	0.96	**20.8**
	High HDI	0.95	28.0	0.58	30.4	0.62	26.7	0.95	13.5	0.95	13.1
	Low HDI	0.97	16.8	0.82	19.4	0.86	24.6	0.97	10.0	0.98	7.8
2022	All	0.96	50.4	0.55	75.3	0.63	62.2	0.96	28.0	0.96	**25.7**
	High HDI	0.95	34.6	0.45	54.7	0.52	43.1	0.95	19.0	0.95	17.2
	Low HDI	0.97	15.9	0.91	20.6	0.86	19.1	0.97	9.0	0.97	8.5

Table S3. Estimated number of migrants to Sweden. Figures are in the scale of 1000. In 2022, we exclude migrants from Ukraine from the analysis since Sweden did not include those protected under the Temporary Protection Directive from its immigration figures; see SI Validation Sweden for more details.

Year	HDI Type	Swedish Government	Raw	Penetration	Raking	Coefficient	Selection
2019	All	108	54	165	144	116	112
	High HDI	67	39	105	84	84	79
	Low HDI	41	15	60	60	32	33
2020	All	77	38	103	135	77	84
	High HDI	54	29	66	89	58	61
	Low HDI	23	9	37	46	19	22
2021	All	86	41	110	114	76	85
	High HDI	58	30	69	65	55	61
	Low HDI	28	11	40	49	21	24
2022	All	97	48	142	104	106	99
	High HDI	67	34	96	61	75	69
	Low HDI	30	14	46	44	32	31

Table S4. Performance comparison among different weighting methods based on the data from Eurostat. Errors are in thousands of people.

Year	HDI type	Raw		Penetration		Raking		Coefficient		Selection rate	
		r	error	r	error	r	error	r	error	r	error
2019	All	0.92	1269.3	0.89	1274.9	0.90	1239.7	0.92	1107.1	0.93	**1012.7**
	High HDI	0.92	862.5	0.86	924.4	0.91	806.1	0.92	905.7	0.93	818.0
	Low HDI	0.99	406.8	0.93	350.4	0.91	433.5	0.99	201.3	0.99	194.7
2020	All	0.90	891.9	0.84	815.0	0.88	838.9	0.91	642.6	0.91	**637.3**
	High HDI	0.89	680.2	0.81	641.8	0.85	646.1	0.89	511.9	0.90	511.7
	Low HDI	0.94	211.6	0.95	173.2	0.96	192.8	0.94	130.7	0.95	125.6
2021	All	0.87	981.2	0.80	904.5	0.84	855.5	0.87	729.8	0.87	**716.0**
	High HDI	0.84	746.9	0.78	715.8	0.81	669.0	0.85	576.5	0.84	580.3
	Low HDI	0.96	234.3	0.93	188.6	0.94	186.5	0.96	153.3	0.96	135.7
2022	All	0.94	1317.2	0.74	1585.1	0.91	1235.1	0.94	1116.0	0.94	**1032.3**
	High HDI	0.94	970.8	0.71	1295.7	0.90	972.8	0.94	860.5	0.94	786.4
	Low HDI	0.96	346.4	0.92	289.4	0.94	262.3	0.96	255.5	0.96	246.0

Table S5. Correlations for 2019

	New Zealand		Sweden		Eurostat	
	N	Correlation	N	Correlation	N	Correlation
Migrants	166	0.98	176	0.87	3293	0.94
Log(Migrants)	166	0.89	168	0.91	2603	0.88
Proportion of Migrants	166	0.98	176	0.87	3293	0.89
Total Outbound	0	NA	0	NA	179	0.96
Total Inbound	1	NA	1	NA	24	0.99
Net Migration	0	NA	0	NA	24	0.99

Table S6. Correlations for 2020

	New Zealand		Sweden		Eurostat	
	**N**	**Correlation**	**N**	**Correlation**	**N**	**Correlation**
Migrants	158	0.99	176	0.95	2923	0.91
Log(Migrants)	158	0.87	166	0.90	2269	0.87
Proportion of Migrants	158	0.99	176	0.95	2923	0.78
Total Outbound	0	NA	0	NA	179	0.96
Total Inbound	1	NA	1	NA	18	0.96
Net Migration	0	NA	0	NA	18	0.94

Table S7. Correlations for 2021

	New Zealand		Sweden		Eurostat	
	N	Correlation	N	Correlation	N	Correlation
Migrants	146	0.99	176	0.96	2566	0.87
Log(Migrants)	146	0.84	169	0.89	1982	0.88
Proportion of Migrants	146	0.99	176	0.96	2566	0.70
Total Outbound	0	NA	0	NA	179	0.95
Total Inbound	1	NA	1	NA	16	0.98
Net Migration	0	NA	0	NA	16	0.97

Table S8. Correlations for 2022. We exclude migrants from Ukraine to Sweden from these analyses since Sweden did not include those protected under the Temporary Protection Directive from its immigration figures; see SI Validation Sweden for more details.

	New Zealand		Sweden		Eurostat	
	**N**	**Correlation**	**N**	**Correlation**	**N**	**Correlation**
Migrants	150	0.99	177	0.97	2900	0.92
Log(Migrants)	150	0.86	170	0.91	2349	0.88
Proportion of Migrants	150	0.99	177	0.97	2900	0.82
Total Outbound	0	NA	0	NA	178	0.98
Total Inbound	1	NA	1	NA	19	0.98
Net Migration	0	NA	0	NA	19	0.97

Table S9. Inflow and outflow of each country

	2019		2020		2021		2022	
iso2	Outflow	Inflow	Outflow	Inflow	Outflow	Inflow	Outflow	Inflow
AD	7,301	8,390	7,414	7,908	7,446	6,923	7,142	9,685
AE	769,808	1,248,145	803,535	643,752	749,831	1,144,597	633,752	2,151,310
AF	103,530	89,590	91,669	66,438	189,945	60,701	166,065	81,698
AL	85,476	33,450	55,911	34,778	76,694	30,131	113,508	28,662
AM	42,025	52,272	25,998	59,099	65,878	43,459	36,459	103,325
AO	67,818	38,101	46,377	24,005	41,674	30,931	56,405	39,884
AR	233,137	181,051	147,823	112,947	181,623	87,456	215,615	161,802
AT	75,894	103,587	78,738	76,056	66,872	82,422	61,468	154,988
AU	276,380	587,275	238,571	278,379	177,536	170,281	187,387	693,583
AZ	47,205	43,218	52,941	35,082	42,696	39,946	35,634	54,940
BA	60,246	26,869	45,815	30,241	46,173	24,059	58,076	23,016
BB	9,359	8,430	9,098	8,560	9,464	7,905	9,370	8,677
BD	720,011	708,969	415,712	568,457	676,047	620,369	1,391,318	699,291
BE	154,433	156,145	120,559	104,747	87,150	114,017	86,528	178,368
BF	30,641	41,271	27,967	39,397	41,849	44,398	56,131	65,231
BG	76,590	64,826	66,512	72,705	63,037	70,065	69,845	91,452
BH	121,757	99,356	110,101	52,981	107,922	80,020	91,447	154,349
BI	29,736	21,234	23,039	22,637	24,080	27,047	36,260	26,908
BJ	40,837	33,645	35,546	39,874	41,272	33,124	44,742	51,085
BN	26,077	28,325	21,014	16,634	20,570	10,483	30,105	23,008
BO	94,729	75,852	83,439	56,575	97,349	67,379	142,032	60,698
BR	487,368	298,911	222,480	247,998	275,492	226,958	547,945	255,427
BS	12,798	12,414	10,844	9,273	14,689	8,259	14,192	13,480
BT	20,812	13,880	17,531	15,699	13,233	9,345	23,932	10,949
BW	14,382	16,052	12,882	14,520	13,495	12,921	18,379	13,856
BY	35,465	22,849	37,073	22,561	55,299	17,779	78,686	31,644
BZ	10,786	9,825	10,275	9,020	11,515	8,204	12,071	8,981
CA	231,918	749,815	204,070	351,121	196,009	533,336	210,034	1,113,926
CD	75,747	81,033	54,918	55,883	64,357	57,956	86,923	60,998
CF	18,643	19,394	18,840	14,573	15,930	11,280	13,119	13,067
CG	30,854	21,794	23,936	16,532	27,358	20,452	31,037	22,611
CH	102,644	146,245	93,016	110,440	88,937	117,810	87,892	202,667
CI	92,683	68,406	80,684	53,943	92,956	79,933	120,966	99,858
CL	126,265	294,280	105,161	116,168	137,304	284,440	169,610	383,607
CM	79,813	40,463	46,436	36,322	65,176	38,529	89,662	42,586
CO	476,970	991,213	312,750	442,357	517,252	550,820	1,119,344	366,085
CR	50,693	58,578	47,433	37,343	55,813	55,487	70,084	69,725
CV	17,506	9,257	13,578	8,467	14,342	8,266	25,302	8,317
CY	33,205	60,474	32,713	42,950	34,584	52,933	37,808	91,806
CZ	58,262	83,238	60,055	64,729	47,025	79,446	50,780	270,575
DE	527,011	756,908	436,989	556,905	460,019	599,642	463,342	1,345,919
DJ	15,252	11,134	13,959	11,550	21,493	8,404	29,317	7,476
DK	38,775	58,907	33,420	50,384	31,940	53,177	31,932	83,045
DO	119,376	93,491	76,814	84,215	105,066	105,580	169,978	110,224
DZ	140,366	85,849	124,884	78,264	121,219	60,543	170,152	67,476
EC	159,954	202,062	128,684	91,476	235,880	113,147	287,348	103,600
EE	13,461	21,004	12,763	17,463	11,664	18,432	13,465	44,908
EG	604,339	505,476	343,670	586,969	481,975	538,491	914,587	466,232
ER	20,487	6,809	10,279	6,755	7,687	6,521	7,251	6,513
ES	305,333	768,788	286,001	423,347	294,047	452,547	280,207	1,026,335
ET	67,401	133,596	56,158	95,763	76,229	120,487	102,422	130,403
FI	26,387	38,004	21,782	32,973	21,208	32,458	21,227	72,424
FJ	17,469	11,140	13,396	12,126	12,026	8,774	24,394	10,389
FM	8,230	9,657	8,606	7,280	8,786	6,061	8,844	6,709
FR	364,592	594,973	280,533	456,177	266,791	465,212	302,363	700,157

Continued on next page

Table S9. Inflow and outflow of each country

	2019		2020		2021		2022	
iso2	Outflow	Inflow	Outflow	Inflow	Outflow	Inflow	Outflow	Inflow
GA	34,086	21,891	24,751	18,234	31,164	19,454	34,247	21,254
GB	670,647	856,989	629,902	658,333	470,148	845,371	442,728	1,336,265
GD	8,790	7,034	9,025	7,298	7,424	7,162	7,433	7,652
GE	65,832	54,661	41,642	52,556	62,183	57,104	95,827	142,900
GH	87,619	84,712	58,654	76,993	85,994	86,889	127,255	97,907
GM	18,504	22,747	17,399	16,810	21,008	20,642	25,450	22,918
GN	62,463	54,171	46,123	41,460	66,017	70,647	85,691	77,770
GQ	11,128	16,634	13,523	11,028	14,384	10,483	15,482	11,216
GR	108,528	105,284	95,941	84,398	119,589	66,556	128,331	87,391
GT	162,067	60,549	81,091	52,734	196,080	57,821	282,674	59,230
GW	14,917	9,507	13,082	12,302	19,486	16,716	22,865	19,837
GY	15,633	15,840	12,149	12,252	15,195	14,080	17,386	12,721
HK	141,175	137,023	137,462	82,873	175,957	80,291	227,873	107,926
HN	173,028	37,383	59,554	35,671	175,243	37,907	208,427	38,202
HR	41,568	34,962	35,532	30,488	32,450	30,355	31,388	54,355
HT	82,898	27,898	64,849	26,556	95,868	36,763	107,673	45,577
HU	56,915	84,142	48,943	75,922	44,967	66,566	57,382	93,763
ID	596,395	748,250	251,159	650,273	206,979	619,149	533,239	574,464
IE	77,893	120,989	67,511	80,212	64,217	81,718	73,317	190,421
IL	79,346	87,826	57,908	53,830	64,564	59,696	65,442	127,439
IN	2,520,754	1,544,878	1,329,500	1,654,110	1,638,248	1,684,086	3,429,268	1,371,867
IQ	112,660	165,750	121,287	123,981	107,685	135,884	111,173	167,685
IS	9,879	13,509	9,591	12,235	8,753	12,312	8,373	17,258
IT	405,085	369,670	300,536	315,529	274,637	347,466	279,804	555,478
JM	38,397	17,443	25,464	16,686	40,516	13,721	55,940	14,104
JO	202,509	101,970	151,627	74,178	152,641	121,104	150,489	111,333
JP	438,531	568,919	349,403	309,346	356,541	246,772	464,053	622,060
KE	100,905	67,724	72,441	58,121	111,218	79,081	169,873	85,134
KG	39,752	39,059	35,969	45,305	62,253	66,340	28,856	89,651
KH	93,454	173,925	82,943	194,430	78,477	173,421	196,466	218,971
KI	6,670	9,143	8,727	7,348	7,002	6,906	7,290	7,072
KM	12,782	10,415	10,863	9,143	11,266	9,912	13,428	10,609
KR	345,074	353,549	278,257	209,655	251,523	146,349	272,984	319,973
KW	312,143	340,821	360,242	116,791	308,802	113,502	286,569	533,330
KZ	55,445	32,515	51,387	32,202	48,786	34,870	42,802	57,501
LA	62,089	65,198	54,607	104,655	83,579	82,190	216,912	81,522
LB	204,157	84,951	234,975	52,910	221,039	67,597	187,912	97,156
LC	8,017	6,664	8,243	7,369	7,900	7,091	8,117	6,774
LK	122,941	108,177	66,810	81,179	83,407	100,003	275,661	78,997
LR	18,944	15,289	16,918	12,682	22,409	15,512	23,552	18,270
LS	20,638	17,119	22,808	16,465	45,749	15,615	57,473	24,316
LT	31,245	37,509	24,149	40,801	19,688	40,118	23,330	69,217
LU	17,241	25,942	17,444	20,145	15,777	20,921	16,379	28,332
LV	21,469	22,560	19,860	21,307	17,715	17,245	21,066	34,189
LY	79,425	110,921	79,393	89,595	95,073	168,174	129,612	177,302
MA	239,073	135,912	185,550	130,713	207,872	121,452	278,507	121,802
MD	66,040	38,669	54,829	51,937	67,960	45,912	89,714	55,291
ME	14,351	13,752	14,615	13,474	12,806	11,695	12,817	28,309
MG	21,056	16,257	17,198	13,543	16,114	11,796	24,865	15,121
MK	35,171	17,390	27,351	18,332	28,926	15,696	41,734	14,797
ML	49,382	60,528	45,713	51,641	52,466	65,939	67,182	70,034
MM	247,327	292,821	150,215	354,655	259,100	121,694	954,028	151,995
MN	39,405	28,897	22,975	30,543	16,988	35,027	45,643	27,197
MO	24,082	27,491	24,859	16,614	20,945	9,843	27,570	9,265
MR	28,853	25,881	26,014	21,840	28,993	25,645	34,264	27,899

Continued on next page

Table S9. Inflow and outflow of each country

	2019		2020		2021		2022	
iso2	Outflow	Inflow	Outflow	Inflow	Outflow	Inflow	Outflow	Inflow
MT	16,529	33,435	18,589	22,666	18,497	19,710	17,857	47,200
MU	22,716	25,218	18,170	16,377	21,610	12,223	28,568	21,960
MV	25,973	30,891	37,748	15,234	22,550	20,145	20,642	38,384
MW	36,791	29,157	23,924	25,068	25,942	32,807	43,313	33,674
MX	535,548	411,515	404,686	353,089	704,209	323,741	1,164,919	330,249
MY	926,923	591,865	832,539	299,361	683,455	154,379	659,573	802,993
MZ	40,836	32,534	37,925	26,657	48,312	29,798	59,238	37,773
NA	14,057	13,134	12,700	11,146	11,837	10,463	13,875	10,897
NE	19,426	26,083	19,030	26,443	23,324	33,046	25,670	41,490
NG	202,936	163,859	140,807	152,620	203,680	149,229	329,187	204,944
NI	93,734	41,890	42,787	48,802	97,335	50,992	261,853	47,478
NL	123,194	190,045	116,640	141,624	106,307	162,183	109,465	271,156
NO	42,603	56,454	34,938	47,086	33,575	45,569	30,832	84,883
NP	428,407	499,772	234,822	482,275	366,172	410,855	911,782	409,404
NZ	85,457	149,261	60,521	100,429	68,423	56,846	87,715	102,305
OM	274,388	215,900	252,905	98,113	302,590	141,350	215,167	354,919
PA	42,882	44,852	41,986	25,528	48,968	29,552	59,879	36,537
PE	250,451	352,229	168,587	135,342	270,782	197,129	507,368	169,187
PG	13,631	12,933	14,860	10,085	11,495	9,328	13,512	11,329
PH	1,113,419	960,591	457,370	752,808	632,137	639,143	1,119,019	794,163
PK	704,808	667,201	414,645	616,570	392,041	623,130	1,413,852	485,986
PL	198,095	286,470	188,080	268,028	146,325	337,158	211,197	868,433
PT	96,512	274,785	91,092	160,892	95,237	166,035	99,885	413,718
PY	64,333	64,973	52,160	47,704	40,667	52,365	98,079	40,771
QA	253,140	351,880	206,886	137,173	189,537	331,632	175,483	549,691
RO	249,475	232,758	192,241	253,355	178,711	231,180	226,445	229,828
RS	61,651	48,025	47,983	48,752	47,008	42,233	56,930	58,146
RU	329,790	233,024	306,391	145,638	335,660	301,118	947,587	82,625
RW	32,520	27,748	27,939	21,730	23,723	20,930	28,377	21,490
SA	2,032,176	1,599,367	1,666,477	840,627	1,882,151	1,207,862	1,715,220	2,798,137
SB	9,325	7,611	9,348	7,714	8,396	7,519	9,320	7,868
SD	206,353	131,297	175,304	101,805	297,307	132,590	337,278	166,354
SE	72,617	113,830	60,441	86,496	60,611	85,989	65,710	126,235
SG	204,891	271,327	205,474	250,163	175,723	138,652	257,990	343,202
SI	17,517	27,267	17,537	21,653	15,623	20,846	15,204	30,068
SK	37,813	50,959	33,983	46,036	34,970	33,985	37,733	64,949
SL	17,927	15,443	15,940	14,322	24,110	16,610	30,365	18,165
SN	89,153	89,900	70,740	61,911	92,053	73,935	105,179	84,726
SR	12,929	11,682	12,790	9,609	13,591	8,499	14,368	9,975
SS	28,860	50,026	31,488	44,168	27,686	57,511	34,086	55,336
ST	9,898	7,167	9,423	7,150	9,868	6,929	14,898	6,917
SV	102,040	33,920	43,728	31,088	90,663	33,287	135,121	29,706
SY	140,730	311,007	166,373	175,655	235,297	149,338	355,909	139,160
SZ	12,421	13,474	12,730	14,210	15,023	12,345	17,884	13,636
TD	19,422	36,916	19,123	26,775	22,791	30,204	27,492	38,324
TG	29,408	26,391	23,270	26,560	26,981	30,103	32,773	44,024
TH	571,133	416,558	636,965	352,336	378,601	363,565	505,074	1,114,982
TJ	24,931	30,072	17,493	28,598	67,344	38,539	33,723	66,031
TL	14,567	26,265	9,405	19,996	8,000	28,818	14,681	31,199
TM	8,728	8,735	9,103	7,906	7,849	7,528	8,818	6,859
TN	100,856	58,415	86,368	54,297	102,159	50,185	176,621	45,221
TO	9,199	7,928	9,645	7,726	9,638	7,648	10,194	7,884
TR	461,574	313,810	279,334	323,111	307,931	354,659	521,337	359,872
TT	17,613	29,051	13,876	20,202	19,327	14,971	22,602	15,685
TW	305,644	306,171	198,971	222,989	179,397	148,069	266,064	274,046

Continued on next page

Table S9. Inflow and outflow of each country

	2019		2020		2021		2022	
**iso2**	**Outflow**	**Inflow**	**Outflow**	**Inflow**	**Outflow**	**Inflow**	**Outflow**	**Inflow**
TZ	41,725	39,506	38,748	36,113	36,030	36,625	38,295	37,931
UA	205,591	131,064	164,592	170,622	277,814	96,964	2,402,184	66,573
UG	106,646	62,482	78,136	57,890	146,520	54,802	143,658	68,934
US	1,242,505	2,283,893	980,507	1,264,227	806,207	2,592,038	841,150	4,109,309
UY	29,405	28,563	21,256	22,669	28,117	19,700	29,083	22,552
UZ	37,663	73,287	28,278	65,645	68,982	88,047	49,060	127,466
VC	7,688	7,008	7,319	7,461	7,075	6,514	7,541	6,683
VE	1,701,436	221,880	502,467	234,669	809,974	343,755	777,440	560,362
VN	658,292	523,981	400,985	297,295	334,627	320,131	647,023	539,049
VU	8,160	6,952	9,521	8,123	10,613	7,626	9,532	8,634
WS	7,511	8,160	9,818	8,220	9,496	7,201	11,879	8,580
XK	35,189	19,342	31,446	14,550	34,511	14,235	49,903	14,124
YE	86,237	124,178	58,452	88,148	120,317	101,771	172,619	94,908
ZA	200,864	194,751	152,393	149,340	177,802	181,685	276,584	220,776
ZM	22,016	30,454	22,893	24,468	22,263	23,560	27,844	27,207
ZW	71,874	39,165	53,204	38,031	51,055	43,564	62,530	70,722

References

1. H Sahai, M Bailey, Social networks and spatial mobility: Evidence from facebook in india (2022).
2. G Chi, B State, JE Blumenstock, L Adamic, Who ties the world together? evidence from a large online social network in *Complex Networks and Their Applications VIII*, eds. H Cherifi, S Gaito, JF Mendes, E Moro, LM Rocha. (Springer International Publishing) Vol. 882, pp. 451–465 (2020).
3. A Herdağdelen, B State, L Adamic, W Mason, The social ties of immigrant communities in the united states in *Proceedings of the 8th ACM Conference on Web Science*, WebSci '16. (ACM), pp. 78–84 (2016).
4. F Willekens, Monitoring international migration flows in europe: Towards a statistical data base combining data from different sources. *Eur. J. Popul.* **10**, 1–42 (1994).
5. F Willekens, Evidence-based monitoring of international migration flows in europe. *J. Off. Stat.* **35**, 231–277 (2019).
6. Stats nz tatauranga aotearoa, international migration. https://www.stats.govt.nz/information-releases/international-migration-june-2022/ (2022).
7. G Hugo, J Wall, M Young, Migration in australia and new zealand. *Int. handbook migration population distribution* pp. 333–370 (2016).
8. GJ Abel, JE Cohen, Bilateral international migration flow estimates for 200 countries. *Sci. Data* **6**, 82 (2019).
9. G Chi, F Lin, G Chi, J Blumenstock, A general approach to detecting migration events in digital trace data. *PLOS ONE* **15**, e0239408 (2020).
10. A Gabadinho, G Ritschard, M Studer, NS Müller, Indice de complexité pour le tri et la comparaison de séquences catégorielles. in *EGC*. pp. 61–66 (2010).
11. B Nowok, F Willekens, A probabilistic framework for harmonisation of migration statistics: Harmonisation of migration statistics. *Population, Space Place* **17**, 521–533 (2011).
12. X Lu, et al., Unveiling hidden migration and mobility patterns in climate stressed regions: A longitudinal study of six million anonymous mobile phone users in bangladesh. *Glob. Environ. Chang.* **38**, 1–7 (2016).
13. E Zagheni, VRK Garimella, I Weber, B State, Inferring international and internal migration patterns from twitter data in *Proceedings of the 23rd International Conference on World Wide Web - WWW '14 Companion.* (ACM Press), pp. 439–444 (2014).
14. JE Blumenstock, G Chi, X Tan, Migration and the Value of Social Networks. *The Rev. Econ. Stud.* p. rdad113 (2023).
15. A Wiśniowski, et al., Utilising expert opinion to improve the measurement of international migration in europe. *J. Off. Stat.* **29**, 583–607 (2013).
16. MA Clemens, , et al., Does development reduce migration. *Int. handbook on migration economic development* **152** (2014).
17. J Poushter, C Bishop, H Chwe, Social media use continues to rise in developing countries but plateaus across developed ones. *Pew research center* **22**, 2–19 (2018).
18. T Lumley, survey: analysis of complex survey samples (2023) R package version 4.2.
19. C Dwork, A Roth, The algorithmic foundations of differential privacy. *Foundations Trends Theor. Comput. Sci.* **9**, 211–407 (2014).
20. B Balle, YX Wang, Improving the gaussian mechanism for differential privacy: Analytical calibration and optimal denoising in *Proceedings of the 35th International Conference on Machine Learning.* (PMLR), pp. 394–403 (2018).
21. J Dong, A Roth, WJ Su, Gaussian differential privacy. *J. Royal Stat. Soc. Ser. B: Stat. Methodol.* **84**, 3–37 (2022).

22. D Doan, M Dornan, J Doyle, K Petrou, Migration and labor mobility from pacific island countries. *Backgr. Pap. for World Dev. Rep.* (2023).
23. T Fic, Migration from bulgaria and romania to the UK. *Natl. Inst. Econ. Rev.* **224**, F4–F7 (2013).
24. D Pavić, I Ivanović, Razlike u prikupljanju migracijskih podataka: usporedba hrvatske i odabranih europskih zemalja. *Migr. Ethn. Themes* **35**, 7–32 (2019).
25. GJ Abel, JE Cohen, Bilateral international migration flow estimates updated and refined by sex. *Sci. Data* **9**, 173 (2022).
26. JJ Azose, AE Raftery, Estimation of emigration, return migration, and transit migration between all pairs of countries. *Proc. Natl. Acad. Sci.* **116**, 116–122 (2019).
27. K Munshi, Networks in the modern economy: Mexican migrants in the u. s. labor market. *The Q. J. Econ.* **118**, 549–599 (2003).
28. L Ryan, A D'Angelo, Changing times: Migrants' social network analysis and the challenges of longitudinal research. *Soc. Networks* **53**, 148–158 (2018).
29. L Ryan, Migrants' social networks and weak ties: Accessing resources and constructing relationships post-migration. *The Sociol. Rev.* **59**, 707–724 (2011).
30. S Bertoli, I Ruyssen, Networks and migrants' intended destination. *J. Econ. Geogr.* **18**, 705–728 (2018).
31. M Bailey, R Cao, T Kuchler, J Stroebel, A Wong, Social connectedness: Measurement, determinants, and effects. *J. Econ. Perspectives* **32**, 259–80 (2018).
